# What We Think Others Think and Do About Climate Change: A Multicountry Test of Pluralistic Ignorance and Public-Consensus Messaging

**DOI:** 10.1177/09567976251335585

**Published:** 2025-05-22

**Authors:** Sandra J. Geiger, Jana K. Köhler, Zenith N. C. Delabrida, Karla A. Garduño-Realivazquez, Christian A. P. Haugestad, Hirotaka Imada, Aishwarya Iyer, Carya Maharja, Daniel C. Mann, Michalina Marczak, Olivia Melville, Sari R. R. Nijssen, Nattavudh Powdthavee, Radisti A. Praptiwi, Gargi Ranade, Claudio D. Rosa, Valeria Vitale, Małgorzata Winkowska, Lei Zhang, Mathew P. White

**Affiliations:** 1 Environmental Psychology Unit, Department of Cognition, Emotion, and Methods in Psychology, Faculty of Psychology, University of Vienna, Austria; 2 Andlinger Center for Energy and the Environment, Princeton University, USA; 3 Department of Psychology, Federal University of Sergipe, Brazil; 4 Department of Accounting, University of Sonora, Mexico; 5 Department of Psychology, University of Oslo, Norway; 6 Research Institute for Future Design, Kochi University of Technology, Japan; 7 Department of Psychology, Christ University, India; 8 Yayasan Puspa Hanuman Indonesia, Indonesia; 9 School of Psychology, University of Plymouth, United Kingdom; 10 Konrad Lorenz Institute of Ethology, University of Veterinary Medicine Vienna, Austria; 11 School of Humanities and Social Sciences, University of St. Gallen, Switzerland; 12 Department of Biology, University of Victoria, Canada; 13 Department of Economics, Nanyang Technological University, Singapore; 14 Research Center for Ecology and Ethnobiology, National Research and Innovation Agency (BRIN), Indonesia; 15 The Shallow End Collective, Bangalore, India; 16 Development and Environment, State University of Santa Cruz, Brazil; 17 Department of Physical Education, Federal Institute of Education, Science and Technology of Northern Minas Gerais (IFNMG), Brazil; 18 Department of Psychology of Developmental and Socialization Processes, Sapienza University of Rome, Italy; 19 Department of Ecology and Environmental Science, Umeå University, Sweden; 20 Centre for Human Brain Health, School of Psychology, University of Birmingham, United Kingdom; 21 Institute for Mental Health, School of Psychology, University of Birmingham, United Kingdom; 22 Center for Developmental Science, School of Psychology, University of Birmingham, United Kingdom; 23 Social, Cognitive and Affective Neuroscience Unit, Department of Cognition, Emotion, and Methods in Psychology, Faculty of Psychology, University of Vienna, Austria; 24 Cognitive Science Hub, University of Vienna, Austria; 25 Department of Clinical and Health Psychology, Faculty of Psychology, University of Vienna, Austria

**Keywords:** climate change, pluralistic ignorance, social norm, cultural tightness-looseness, cross-country generalizability

## Abstract

Most people believe in human-caused climate change, yet this public consensus can be collectively underestimated (*pluralistic ignorance*). Across two studies using primary data (*n* = 3,653 adult participants; 11 countries) and secondary data (*n*s = 60,230 and 22,496 adult participants; 55 countries), we tested (a) the generalizability of pluralistic ignorance about climate-change beliefs, (b) the effects of a public-consensus intervention on climate action, and (c) the possibility that cultural tightness-looseness might serve as a country-level predictor of pluralistic ignorance. In Study 1, people across 11 countries underestimated the prevalence of proclimate views by at least 7.5% in Indonesia (90% credible interval, or CrI = [5.0, 10.1]), and up to 20.8% in Brazil (90% CrI = [18.2, 23.4]. Providing information about the actual public consensus on climate change was largely ineffective, except for a slight increase in willingness to express one’s proclimate opinion, δ = 0.05 (90% CrI = [−0.02, 0.11]). In Study 2, pluralistic ignorance about willingness to contribute financially to fight climate change was slightly more pronounced in looser than tighter cultures, highlighting the particular need for pluralistic-ignorance research in these countries.

Most people worldwide believe climate change is happening and is, at least in part, human-caused (YouGov Cambridge, 2020). However, this public consensus can be collectively underestimated—an example of a phenomenon known as *pluralistic ignorance* (Korte, 1972; Prentice & Miller, 1993). Arguably, the most nuanced assessment of pluralistic ignorance in the context of climate-change beliefs comes from an Australian study (Leviston et al., 2013). Although Australians thought a minority of Australians believed in natural or human-caused climate change, both of these views were (near) majority views. On the flip side, Australians, especially climate skeptics, vastly overestimated the prevalence of minority views that climate change was not happening (*false consensus*; Marks & Miller, 1987; Ross et al., 1977).

An open question is to what extent these pluralistic-ignorance effects generalize across time and place. Although the original study (2013) showed that around 40% of Australians believed in natural causes of climate change, such beliefs are nowadays held by a small minority in Australia and worldwide (5%–18%; YouGov Cambridge, 2020). These lower levels of climate skepticism are expected to be over- rather than underestimated, given that people generally overestimate small proportions and underestimate large ones (Landy et al., 2018). In terms of place, Mildenberger and Tingley (2019) found pluralistic ignorance about climate-change beliefs in China and the United States, supporting generalizability beyond Australia. More recent work has also suggested pluralistic ignorance about climate-policy support in the United States (Sparkman et al., 2022) and behavioral intentions to contribute financially to fight climate change in various countries (Andre et al., 2024).^1^ Given that the presence and magnitude of pluralistic ignorance depend on the actual public consensus (Andre et al., 2024), we cannot merely generalize from previously demonstrated pluralistic ignorance in the climate-change domain in some countries to pluralistic ignorance of climate-change beliefs across contexts—an important motivation for the current work.

Apart from the actual consensus, the broader cultural context may contribute to pluralistic ignorance. Compared with tight cultures (e.g., China and India), looser cultures (e.g., Australia and Brazil) are characterized by more ambiguous social norms and more tolerance for norm violations (Gelfand et al., 2011, 2021)—resulting in less frequent opportunities for norm clarification and communication (Chen et al., 2022; Mulder, 2008; Sarin et al., 2021). In the context of climate change, looser cultures and their greater tolerance of norm-deviant behaviors and (by extension) opinions may contribute to skeptical minorities being more vocal about their opinions. Because looser cultures deem direct punishment, such as physical confrontation and social ostracism, less appropriate in response to norm violations than tighter cultures do (Eriksson et al., 2021), climate skeptics may expect less punishment for expressing their opinions and may, therefore, be more outspoken about them. According to the *spiral of silence theory* (Noelle-Neumann, 1993), such vocal minorities can foster pluralistic ignorance—the impression that skeptical views on climate change are more prevalent than they are in reality.

Overestimating the prevalence of skeptical views or underestimating the prevalence of proclimate views may have adverse consequences. In line with the spiral of silence theory (Noelle-Neumann, 1993), such errors in estimation may cause self-silencing among those who hold proclimate views (Geiger & Swim, 2016), exacerbating the impression that proclimate views are not widely shared and further discouraging societal discourse around climate change. These estimation errors can also hamper support for climate policies (Ban Rohring & Akerlof, 2020; Mildenberger & Tingley, 2019) and undermine climate action (Ballew et al., 2019; Kjeldahl & Hendricks, 2018).

On the bright side, pluralistic ignorance provides an opportunity for simple and scalable interventions (Boon-Falleur et al., 2022) that emphasize the actual public consensus on climate change and promote outcomes related to climate action. According to the *gateway belief model* (van der Linden, 2021; van der Linden et al., 2019), communicating the scientific consensus on climate change—a different form of social consensus—should result in updated, more accurate perceptions that are expected to strengthening nontargeted outcomes, such as proclimate views, climate change worry, and support for public action on climate change. Supporting this notion, empirical evidence showed that scientific-consensus messages increased nontargeted outcomes directly (Goldberg et al., 2022; van Stekelenburg et al., 2022; Većkalov et al., 2024; Vlasceanu et al., 2024) and indirectly through changes in perceptions of the scientific consensus (Kerr & Wilson, 2018; van der Linden et al., 2015; van der Linden et al., 2019). Similarly, messages emphasizing public consensus on climate change can spill over to behavioral intentions, climate-policy support (Bursztyn & Yang, 2021; Nolan, 2011), and nontargeted but related perceptions (e.g., of others’ climate-action support and climate-policy support; Cialdini et al., 1990). For example, Americans were more willing to support climate policies and join a climate-change campaign when they perceived that a majority (vs. minority) of Americans believed in climate change (Ballew et al., 2019). Informing Americans that most Americans are angry about climate inaction boosted climate-change beliefs and policy support (Sabherwal et al., 2021). In line with *social identity theory* (Tajfel & Turner, 2004), such public-consensus interventions are especially effective when people strongly identify with the referent group, and this identification can be more important for message effectiveness than the geographical closeness of the referent group (Cialdini & Jacobson, 2021; Liu et al., 2019; Stok et al., 2014; Xiao et al., 2023).

## Overview of the Present Studies

An overview of the studies’ aims, research questions, and hypotheses can be found in Table 1. In Study 1, we used primary data to conceptually replicate Leviston et al.’s (2013) work (see Supplement A in the Supplemental Material available online) and test whether pluralistic ignorance of climate-change beliefs generalizes across a diverse set of 11 countries (*n* = 3,653; Brazil, Canada, China, Germany, India, Indonesia, Italy, Japan, Mexico, Poland, and Thailand; Aim 1).

**Table 1.** Overview of the Studies’ Aims, Research Questions, and Hypotheses

**Aim 1: Pluralistic ignorance (Study 1)**	
Generalizability of pluralistic ignorance across countries (preregistered)	Hypothesis 1: Individuals underestimate the number of people in their country who believe that climate change is (a) mainly and (b) partly human-caused. Individuals overestimate the number of skeptics in their country—(c) attribution skeptics (i.e., people who believe climate change is happening but is not human-caused) and (d) trend skeptics (i.e., people who believe climate change is not happening).
	Research Question 1: Does pluralistic ignorance (Hypothesis 1a–d) generalize across countries?
	Hypothesis 2: Individuals who believe climate change is (a) mainly or (b) partly human-caused underestimate the prevalence of this opinion in their country the least compared with other belief groups. (c) Attribution skeptics and (d) trend skeptics overestimate the prevalence of this opinion in their country the most compared with other belief groups.
**Aim 2: Public-consensus intervention (Study 1)**	
Effectiveness of the public-consensus intervention (preregistered)	Hypothesis 3: Climate-change believers^a^ in the intervention (vs. control) condition are more willing to express their opinion on climate change.
	Hypothesis 4: Climate-change believers^a^ in the intervention (vs. control) condition (a) are more willing to make changes to their lifestyle to mitigate climate change and (b) expect more fellow citizens to be willing to make at least some changes to their lifestyle to mitigate climate change.
	Hypothesis 5: Climate-change believers^a^ in the intervention (vs. control) condition (a) are more likely to view government action on climate change as a higher priority and (b) are more likely to expect that their fellow citizens view government action on climate change as a high or very high priority.
	Research Question 2: Do climate-change believers in the intervention (vs. control) condition believe more strongly that their country’s citizens can contribute to reducing climate change (i.e., group efficacy beliefs)?
Effectiveness of the public-consensus intervention among different audiences (preregistered)	Hypothesis 6: The effects of the intervention on (a) willingness to make lifestyle changes to mitigate climate change and (b) support for government action on climate change are stronger for climate-change believers with higher rather than lower national identification. Research Question 3: Is the effect of the intervention on group efficacy beliefs stronger for climate-change believers with higher than lower national identification?
**Aim 3: Country-level predictor of pluralistic ignorance (Study 2)**	
Cultural tightness-looseness (not preregistered)	Exploratory Research Question 4: Country-level cultural tightness-looseness predicts pluralistic ignorance about willingness to contribute at least 1% of one’s income to fight climate change, such that looser cultures show more pluralistic ignorance than tighter cultures.

Note: ^a^We focused on climate-change believers because some of the expected effects may differ for climate-change skeptics. For example, willingness to discuss may be higher for climate-change believers after the intervention as they learn their opinion is the majority opinion. In contrast, it may be lower for climate-change skeptics as they learn their opinion is the minority opinion (Geiger & Swim, 2016). However, prior to data collection, we expected few climate-change skeptics in our samples and, thus, insufficient statistical power to test whether individual climate-change beliefs moderate intervention effects.

Going beyond testing the generalizability of pluralistic ignorance, we provide an ecologically valid assessment of whether and for whom an intervention presenting the actual public consensus on climate change could promote factors related to climate action (Aim 2). So far, many public-consensus interventions in the environmental domain have relied on fictitious, nonpolled data (e.g., Cole et al., 2022; Geiger & Swim, 2016; Lu, 2023; Sabherwal et al., 2021). Although this approach is valuable, especially when elucidating mechanisms, it may paint a more optimistic picture of the social consensus/norm than in reality. Given that social-consensus interventions are more effective when prior estimates are less accurate (Lees & Cikara, 2020; van Stekelenburg et al., 2022; Većkalov et al., 2024), interventions based on nonpolled, potentially more optimistic data may inflate intervention effects. To address these shortcomings and increase the external validity of our findings, we used country-specific representative data on the actual climate-change consensus (YouGov Cambridge, 2020).

Building on theoretical considerations and findings from Study 1, we used secondary data from 55 countries in Study 2 (*n* = 60,230, Andre et al., 2024; *n* = 22,496, Eriksson et al., 2021) to explore whether cultural tightness-looseness predicts pluralistic ignorance about others’ proclimate behavioral intentions—specifically, others’ willingness to contribute at least 1% of their household income to fight climate change (Aim 3).

## Research Transparency Statement

### General disclosures

**Conflicts of interest:** The authors declare no conflicts of interest. **Funding:** This research was supported by a preregistration grant from the Leibniz Institute for Psychology (ZPID), an early adopter grant from besample (https://besample.app/), and an internal grant from the Doctoral School in Cognition, Behavior, and Neuroscience at the University of Vienna. **Artificial intelligence:** No artificial-intelligence-assisted technologies were used in this research or the creation of this article. **Ethics:** This research complies with the Declaration of Helsinki (2023), and Study 1 received approval from the ethics board at the University of Vienna (Project No. 00769 and Project No. 00843).

### Study 1 disclosures

**Preregistration:** The research aims/hypotheses, methods, and analysis plan were preregistered on PsychArchives (June 29, 2022; https://doi.org/10.23668/psycharchives.7059) prior to data collection on September 14, 2022. There were major and minor deviations from the preregistration (for details, see Supplement B in the Supplemental Material). **Materials:** All study materials are publicly available (https://doi.org/10.17605/OSF.IO/6W5BD). **Data:** All primary data are publicly available (https://doi.org/10.17605/OSF.IO/R2BYZ). **Analysis scripts:** All analysis scripts are publicly available (https://doi.org/10.17605/OSF.IO/GWEZM). **Computational reproducibility:** The computational reproducibility of the results has been independently confirmed by the journal’s STAR team.

### Study 2 disclosures

**Preregistration:** No aspects of the study were preregistered because we used secondary data and had to access the data to decide whether they could be used for testing the proposed hypothesis. **Materials:** All study materials are publicly available (https://doi.org/10.17605/OSF.IO/N62AD). **Data:** Primary data is not reshared, but all primary data are publicly available via Andre et al. (2024; https://doi.org/10.15185/gccs.1) and Eriksson et al. (2021; https://osf.io/pm5kc/). The combined secondary data set is publicly available (https://doi.org/10.17605/OSF.IO/N73AE). **Analysis scripts:** All analysis scripts are publicly available (https://doi.org/10.17605/OSF.IO/9H756). **Computational reproducibility:** The computational reproducibility of the results has been independently confirmed by the journal’s STAR team.

## Study 1

### Materials and method

#### Participants

Participants were recruited through respondi (https://www.respondi.com/access-panel), an external panel provider certified under ISO 20252, from September 14 to October 13, 2022. We collected cross-quota samples on the basis of age and sex in each of the 11 countries. For inclusion, participants needed to be at least 18 years old, citizens and residents of one of the 11 countries, and proficient in the language in which the survey was administered in the respective country. Participants were compensated according to the panel’s standard criteria, with points that could be redeemed for money, a voucher, or a donation, depending on the participant’s choice.

A total of 8,151 participants started the online survey experiment. Of these, 3,474 were screened out because the quota was already reached (*n* = 3,369; 41.3%) or because they were not eligible for the study (*n* = 105; 1.3%). An additional 533 participants were excluded because they failed the attention check (*n* = 80; 1.0%) or because they completed the survey experiment in under 3 min^2^ (*n* = 453; 5.6%). Moreover, 475 (5.8%) participants did not complete the survey experiment, and 16 (0.2%) were potential bots (reCAPTCHA < 0.5; not preregistered) and thus excluded. These exclusions resulted in an analytic sample of 3,653 participants across 11 countries. In line with population characteristics, participants were, on average, 43.60 years old (*SD* = 15.79; range = 18–85 years), and 50.3% (*n* = 1,839) were female (Table 2). In total, 57.5% (*n* = 2,099) held a university degree, and most participants (76.6%; *n* = 2,797) lived in urban areas.

**Table 2.** Sample and Population Statistics by Country

Weighted cross-quota samples													Population^1^		YouGov Globalism Survey				
Country	*n*	Mean age (*SD*)	Female (%)	University degree (%)	Mean political orientation (*SD*)	Mean national identification (*SD*)	Urban (%)	Mainly human-caused (%)	Partly human-caused (%)	Not human-caused (%)	No climate change (%)	Do not know (%)	University degree (%)^2^	Urban (%)^3^	Mainly human-caused (%)	Partly human-caused (%)	Not human-caused (%)	No climate change (%)	Don’t know (%)
Brazil	331	40.5 (14.8)	51.5	55.5	5.8 (3.2)	9.4 (1.4)	96.6	55.0	33.0	5.0	4.0	3.0	16.5	88	55 ±3.07	33 ±2.90	5 ±1.34	4 ±1.21	3 ±1.05
Canada	329	45.2 (16.1)	49.7	61.1	4.9 (2.4)	8.8 (1.7)	73.7	39.0	44.0	8.0	3.0	6.1	25.8	82	39 ±2.99	44 ±3.04	8 ±1.66	3 ±1.05	7 ±1.57
China	333	43.5 (15.3)	49.0	82.5	5.1 (1.7)	9.0 (1.7)	95.1	24.0	66.7	2.4	2.1	4.8	15.5^4^	64	23 ±2.59	64 ±2.95	5 ±1.34	2 ±0.86	5 ±1.34
Germany	329	48.0 (16.5)	50.0	32.3	5.0 (1.8)	8.0 (2.0)	58.8	31.7	53.2	8.2	2.0	4.9	28.4	78	31 ±2.83	52 ±3.06	8 ±1.66	2 ±0.86	7 ±1.56
India	330	38.0 (14.8)	48.2	81.8	6.3 (2.7)	8.6 (2.7)	80.7	51.0	27.0	11.0	5.0	6.0	12.2	36	51 ±3.00	27 ±2.66	11 ±1.88	5 ±1.31	6 ±1.42
Indonesia	330	39.2 (14.7)	49.2	58.5	6.2 (2.0)	9.5 (1.1)	80.1	31.2	42.6	17.0	3.1	6.1	10.5	58	30 ±2.75	41 ±2.95	18 ±2.30	3 ±1.02	8 ±1.63
Italy	328	47.5 (15.5)	51.9	36.2	4.9 (2.6)	8.4 (2.1)	70.8	47.1	41.0	6.1	2.0	3.7	16.5	72	46 ±2.67	40 ±2.63	6 ±1.27	2 ±0.75	6 ±1.27
Japan	354	48.6 (16.1)	50.3	54.6	5.0 (1.4)	8.6 (1.9)	48.9	36.4	49.5	5.0	2.0	7.1	19.9	92	36 ±2.95	49 ±3.07	5 ±1.34	2 ±0.86	7 ±1.57
Mexico	328	39.5 (14.5)	51.2	66.0	5.7 (2.7)	9.3 (1.6)	90.1	47.8	33.8	13.9	2.0	2.4	17.1	81	48 ±3.06	34 ±2.90	14 ±2.12	2 ±0.86	3 ±1.04
Poland	330	45.5 (16.0)	51.2	44.2	5.6 (2.8)	8.6 (2.2)	74.6	41.6	39.6	7.9	4.0	6.9	23.1^5^	60	42 ±3.06	40 ±3.03	8 ±1.68	4 ±1.21	7 ±1.58
Thailand	331	43.8 (14.5)	51.5	59.3	5.1 (2.3)	8.9 (1.9)	74.8	42.0	37.0	10.0	5.0	6.0	15.6	53	42 ±3.02	37 ±2.96	10 ±1.84	5 ±1.33	6 ±1.45
Totals	3,653	43.6 (15.8)	50.3	57.5	5.4 (2.4)	8.9 (1.9)	76.6	40.6	42.6	8.6	3.1	5.2							

Note: ^1^The population-level data are compiled from different years, countries, and sources, with varying definitions of educational degrees and urban areas. These data, therefore, serve only the purpose of comparisons with the samples in this study. ^2^Percentage of the population (25 years or older) that completed a bachelor’s degree or equivalent, according to data from the World Bank from 2010 to 2020, depending on the country (World Bank, 2022a). ^3^Percentage of the total population living in urban areas, according to data from the World Bank in 2022 (World Bank, 2022b). ^4^Source: Chinese government (2021), https://www.gov.cn/guoqing/2021-05/13/content_5606149.htm. ^5^Source: Statistics Poland (2021), https://stat.gov.pl/spisy-powszechne/nsp-2021/nsp-2021-wyniki-wstepne/ludnosc-wedlug-cech-spolecznych-wyniki-wstepne-nsp-2021,2,1.html. The cross-quota samples regarding age and sex were stratified climate-change beliefs based on the YouGov data. Political orientation values range from 0 (*left*) to 10 (*right*); national-identification values range from 0 (*strongly disagree*) to 10 (*strongly agree*). In some countries, the sample size deviates from the preregistered number (*n* = 330) because (a) a small number of potential bots were excluded (Canada, Germany, Italy, Japan, Mexico), (b) several participants completed the survey at the same time while the quota was just being reached (Brazil, China, Thailand), or the panel provider recruited an additional 10% because of potential bots (Japan). The ± values indicate the margins of error based on the 95% confidence intervals calculated using the R package *moe* (Version 0.9.1; Dahlgren, 2023).

#### Sampling plan

We conducted a priori power analyses on the basis of the original analysis plan (see the preregistration). Because of changes to the preregistered plan, we conducted new power analyses to match the final analyses more closely. Specifically, we ran power simulations based on frequentist fractional logistic regressions in each country. These simulations indicated that 300 participants per country allowed us to reliably detect (*p* = .01 and power ≥ 95%) very small effects of pluralistic ignorance (i.e., a 5 percentage point difference between perceived beliefs of others and actual beliefs). Although previous research coded differences of less than 10 percentage point as accurate when estimating a dichotomous outcome (Carey et al., 2022), we conservatively set the threshold to 5 percentage point because participants in this study estimated five climate-change beliefs—which reduces the effect—and because lower levels of misperceptions are unlikely to be practically relevant. We did not conduct any a priori power analyses for the intervention effects because the available computational resources did not allow for running several thousand Bayesian ordinal regression models. However, as we focus on overall effects across all 11 countries, 3,300 participants should suffice to reliably estimate and detect small but meaningful overall effects (for a similar argument, see Hoogeveen et al., 2022).

#### Country selection

We selected the 11 countries on the basis of availability and geographic spread. We included only countries on the respondi online panel with high-quality public-opinion data about climate change. When selecting countries, we aimed for diversity in terms of actual proclimate consensus (ranging from 71% in Indonesia to 88% in Brazil; see Supplement C in the Supplemental Material), cultural tightness-looseness, and geographic spread, following recent calls to increase national diversity in psychology (Tam & Milfont, 2020). National diversity is pivotal for environmental psychology because climate change affects some countries more than others (Eckstein et al., 2021; King & Harrington, 2018). At the same time, these countries are often understudied in environmental psychology. To draw conclusions about the more global (vs. local) nature of pluralistic ignorance in the context of climate-change beliefs, we paid special attention to the inclusion of countries that are usually understudied but are disproportionately affected by climate change (e.g., India, Japan, and Thailand; Eckstein et al., 2021).

#### Materials

All materials and measures are presented in Table 3. The experimental manipulation consisted of a control and intervention message adapted from past studies on political polarization (Lees & Cikara, 2020; Ruggeri et al., 2021). In the control condition, participants were informed about their previous estimates of the public consensus on climate change: “Previously, you estimated that [x]% of [country citizens] believe that the climate is changing and human activity is partly ([x]%) or mainly ([x]%) responsible.” In the intervention condition, participants were presented with the following additional message and a graphic adapted to each country (Supplement C): “You might be interested to know that a recent survey showed that [x]% of [country citizens] believe that the climate is changing and human activity is partly ([x]%) or mainly ([x]%) responsible.” The distribution of climate-change beliefs was based on real-world data from the 25-country YouGov Globalism survey (YouGov Cambridge, 2020) and thus varied across countries, from 71% of Indonesians to 88% of Brazilians (Supplement C). The YouGov Globalism survey invited a random subsample from an online panel to participate in the survey between July 30 and August 24, 2020. The samples (*n*s = 1,001–1,337 participants) were representative of the country’s adult population (Brazil, Canada, Germany, Italy, Japan, Mexico, and Poland) or online adult population (China, India, Indonesia, and Thailand), at least in terms of age, gender, and region (J. Buckle, personal communication, July 7, 2022).

**Table 3.** Overview of Materials and Measures in Study 1

Construct	Measure	Response categories
National identification (moderator; Van Bavel et al., 2022)	I identify as [nationality]. Being a [nationality] is an important reflection of who I am. To create an overall national identification score, we averaged across the two items (τ_*b*_ = .56, 95% CrI = [.53, .58], BF_10_ *→* ∞).	0 = *strongly disagree*, 5 = *neither agree nor disagree*, 10 = *strongly agree*
Description of climate change (Maibach et al., 2013)	*Climate change* refers to the idea that the world’s average temperature has been increasing over the past 150 years, will increase more in the future, and that the world’s climate will change as a result.	
Climate-change beliefs (outcome; YouGov Cambridge, 2020)	In general, which of the following statements, if any, best describes your view?	1 = *The climate is changing, and human activity is mainly responsible.* 2 = *The climate is changing, and human activity is partly responsible, together with other factor*s. 3 = *The climate is changing but human activity is not responsible at all.* 4 = *The climate is not changing.* 5 = *I don’t know.* 0% = *no one* to 100% = *everyone*
Perceived climate-change beliefs of others (outcome; Leviston et al., 2013; Mildenberger & Tingley, 2019)	What percentage of [country citizens], do you believe, would think the following ways about climate change? Please indicate a number from 0% (*no one*) to 100% (*everyone*) for the following statements such that they sum up to 100%. The climate is changing, and human activity is mainly responsible. The climate is changing, and human activity is partly responsible, together with other factors. The climate is changing but human activity is not responsible at all. The climate is not changing. Don’t know. (Please indicate what percentage of [country citizens] does not know what they think about climate change.	
Pluralistic ignorance (outcome)	Accuracy score (Sargent & Newman, 2021): the difference between an individual’s belief about others’ climate-change beliefs and the percentage of individuals holding the respective climate-change belief in each country. For example, if a Mexican participant estimated that 20% of Mexicans believe the climate is not changing, but only 2% of Mexicans think so, the participant’s score would be +18% (20% – 2%).	
Willingness to express one’s opinion on climate change among fellow citizens (outcome; Geiger & Swim, 2016)	“How willing or unwilling are you to express your opinion on climate change among [country citizens] you don’t know?”	1 = *not at all willing* to 7 = *very willing*
Expectations about others’ willingness to make lifestyle changes and support government action (outcomes)	For the following question, please consider what [country citizens] think about climate change. What percentage of [country citizens], do you believe, would be willing to make the following extent of changes to how they live and work to help reduce the potential effects of climate change? • No [changes] or a few changes • Some [changes] or a lot of changes Please indicate a number from 0% (no one) to 100% (*everyone*).For the following question, please consider what [country citizens] think about climate change. What percentage of [country citizens], do you believe, think climate change should be a low/medium or high/very high priority of the government of [country]? • Low or medium priority • High or very high priority Please indicate a number from 0% (*no one*) to 100% (*everyone*).	0% = *no one* to 100% = *everyone*
Willingness to make lifestyle changes and support government action (outcomes; Bell et al., 2021; Leiserowitz et al., 2021)	How much, if anything, would you be willing to change about how you live and work to help reduce the potential effects of climate change? Do you think climate change should be a low, medium, high, or very high priority for the government of [country]?	*No changes at all, a few changes, some changes*, and *a lot of changes* *Low, medium, high*, and *very high*
*Perceived group efficacy* (outcome)	To what extent do you think that [country citizens] can jointly prevent the negative consequences of climate change? We selected one of the three items from the original scale on the basis of face validity and domain coverage because of the high reliability of Cronbach’s alpha (α = .94) in past research (van Zomeren et al., 2010) and thus the repetitiveness of the full scale. Using the Spearman-Brown prediction formula (Dahlke & Wiernik, 2019), the reliability of this shortened instrument was estimated to be very good (Cronbach’s α = .84).	1 = *not at all* to 7 = *very much*, with two additional options— *Don’t know* and *I don’t believe in (human-caused) climate change*
Attention check	With this question, we would like to ensure that participants pay attention. Please select the option “Red” from the list below.	*Blue*, *red*, *yellow*, *green*, and *white*
Demographic information (control variables and weighting)	Age, sex, citizenship, country of residence, urban/rural region, highest completed level of education, political orientation	Age: continuous (in years) Sex: female, male Citizenship: yes, no Country of residence: yes, no Urban/rural region: urban, rural, don’t know Highest completed level of education: seven categories from 0 *= no formal education* to 7 *= doctoral degree*, adapted to each country Political orientation: 0 = *left* to 10 *= right*

Note: BF = Bayes factor. CrI = credible interval.

### Procedure

The study was approved by the Institutional Review Board at the University of Vienna (Project No. 00769 and Project No. 00843) and translated into one of the local languages in each country using a standard forward-and-back translation approach (Forscher et al., 2020; Jarke et al., 2022; see Supplement D in the Supplemental Material). After providing informed consent, participants indicated their age, sex, citizenship, country of residence, and national identification. Participants who did not consent or were not citizens and residents of one of the 11 countries were redirected to the end of the survey. Subsequently, participants read a short description of climate change before indicating their personal climate-change beliefs and perceptions of others’ climate-change beliefs in counterbalanced order across participants.^3^ The order of the five belief categories was also counterbalanced across participants—either increasing or decreasing (from *not happening* to *mainly human-caused* or vice versa, with *don’t know* as a fixed fifth option). Participants were then randomly but evenly assigned (by the survey program Qualtrics) to the control or intervention condition; they then completed the six outcome measures and an attention check and provided their remaining demographics (education, political orientation, and urbanicity). The median time to complete the survey was 6.4 min.

#### Data analysis

All analyses were conducted in R (Version 4.1.0; R Core Team, 2021).

##### Poststratification weighting

We used poststratification raking (R package *anesrake*, Version 0.80; Pasek, 2018) to align the distribution of climate-change beliefs in the samples with the YouGov data (2020), ensuring adequate representation of all climate-change belief groups. Following the European Social Survey (Vehovar et al., 2014), we trimmed weights at 4.0 in six countries (Canada, China, Germany, Indonesia, Italy, and Mexico) to reduce the effect of outliers. For all other five countries, weight trimming was not necessary.

##### Statistical models

We used a Bayesian approach to data analysis whenever possible to quantify the relative support for or against any hypothesis and to communicate this gradual evidence in an easy-to-understand way (Quintana & Williams, 2018). We used the R packages *brms* (Version 2.16.3; Bürkner, 2018) and *RStan* (Version 2.21.3; Stan Development Team, 2022). To test pluralistic ignorance effects (Hypothesis 1a–d and Research Question 1), we fitted Bayesian multilevel zero-one-inflated regression models, including weights (level 1: participants, level 2: countries), that account for the doubly bounded outcomes, namely perceived climate-change beliefs (0–100%). Priors were weakly informative defaults, with α, γ ~ logistic(0, 1) for the probability of an observation being a 0 or 1 (α) and the probability of an observation being a 1 given that it is either 0 or 1 (γ), μ, φ ~ Student *t*(3, 0, 2.5) for mean (μ) and precision (φ) parameter of the beta distribution, as well as σ ~ Student *t*(3, 0, 2.5) for all variance parameters.

To test intervention effects, we estimated Bayesian multilevel cumulative probit regressions for ordinal outcomes (Hypotheses 3, 4a, and 5a and Research Question 2) and frequentist fractional logistic regressions for percentage data (Hypotheses 4b and 5b), including country as a fixed predictor. We controlled for age, sex, education, and political orientation if they were associated with the outcome. For the cumulative probit models, we used uniform priors on the threshold parameters and weakly informative priors for the predictor coefficients, *b* ~ Normal(0, 10), and all variances, σ ~ HalfNormal(0, 1). Decision criteria regarding the hypotheses and research questions include posterior distributions, credible intervals (CrIs), and Bayes factors (BFs; see Supplement E).

### Results

#### Pluralistic ignorance across countries (Aim 1)

**Fig. 1.**
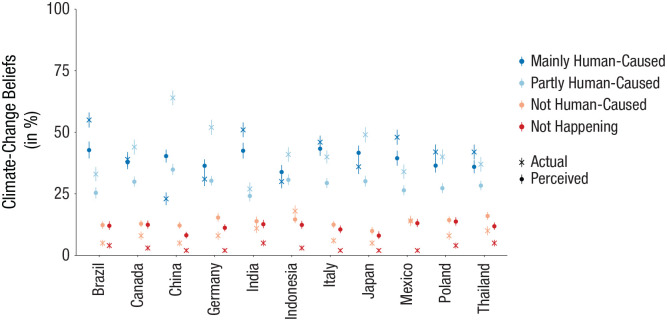
Actual and perceived prevalence (in %) of different climate-change belief types by country. Colors indicate the four types of climate-change beliefs (mainly human-caused, partly human-caused, not human-caused, and not happening). The fifth type, *don’t know*, is not displayed. The crosses indicate the actual percentage of participants who endorsed each of the beliefs in the YouGov survey; the error bars indicate the margins of error based on 95% confidence intervals, calculated using the R package *moe* (Version 0.9.1; Dahlgren, 2023). The dots indicate posterior means of participants’ estimates of what percentage of people in their country endorsed each of the four climate-change beliefs. These posterior means are based on Bayesian multilevel zero-one-inflated regression models. The error bars indicate 95% equal-tailed credible intervals, calculated using the R package *bayestestR* (Version 0.13.1; Makowski et al., 2019). Countries are ordered alphabetically. Sample sizes range from 328 in Italy and Mexico to 354 in Japan (total *n* = 3,653). Across countries, people relatively consistently underestimated proclimate views (dark and light blue) and consistently overestimated skeptical views (dark and light red).

We found reliable differences in the expected direction between the actual and perceived consensus on human-caused climate change—indicating pluralistic ignorance. The prevalence of proclimate views was relatively consistently underestimated compared with the actual beliefs (Fig. 1). Consistent with Hypothesis 1a, we found at least moderate evidence in seven countries that people underestimated the prevalence of views that climate change was mainly human-caused (range: −1.2%, 90% CrI = [−3.5%, 1.2%], BF_-+_ = 3.78 in Canada; −12.3%, 90% CrI = [−15.1%, −9.3%], BF_-+_ → ∞ in Brazil). However, we also found extremely strong evidence that people in the remaining four countries overestimated the size of this group (range: 3.8%, 90% CrI = [1.5%, 6.2%], BF_-+_ = 311.53 in Indonesia; 17.3%, 90% CrI = [15.2%, 19.6%], BF_-+_ → ∞ in China). Supporting Hypothesis 1b, people across all 11 countries underestimated the prevalence of views that climate change was partly human-caused (range: −2.8%, 90% CrI = [−4.7%, 1.0%], BF_-+_ = 195.08 in India; −29.1%, 90% CrI = [−31.0%, −27.2%], BF_-+_ → ∞ in China), with extremely strong evidence. On the other hand, the prevalence of skeptical views was consistently overestimated across countries (Fig. 1). Supporting Hypothesis 1c, we found extremely strong evidence that people overestimated the prevalence of views in all countries that climate change was not human-caused (range: 2.9%, 90% CrI = [1.5%, 4.4%], BF_-+_ = 5,453.55 in India; 7.4%, 90% CrI = [6.0%, 8.9%], BF_-+_ → ∞ in Germany) except Indonesia and Mexico. Consistent with Hypothesis 1d, people across all 11 countries overestimated the prevalence of views that climate change was not happening, with extremely strong evidence (range: 6.2%, 90% CrI = [5.1%, 7.3%], BF_-+_ → ∞ in China; 11.1%, 90% CrI = [9.7%, 12.5%], BF_-+_ → ∞ in Mexico).

**Fig. 2.**
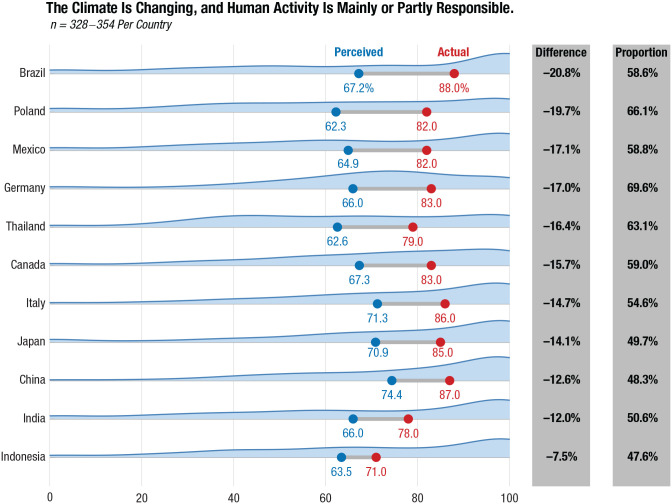
Actual (red) and perceived (blue) prevalence (in %) of proclimate beliefs (mainly human-caused or partly human-caused) by country. The blue area represents the distribution of the perceived proportion of climate-change believers per country. The blue dot represents the posterior mean of the perceived proportion of climate-change believers per country, based on multilevel zero-one-inflated regression models. The red dot represents the actual proportion of climate-change believers based on the YouGov data. The difference column represents the difference between the perceived and actual proportion per country; negative values indicate that people, on average, underestimated the proportion of climate-change believers in their country. The proportion column indicates the proportion of participants per country who underestimated the consensus on climate change in their country. Countries are ordered by the magnitude of pluralistic ignorance, from largest to smallest.

When collapsing belief types into two categories (believers vs. nonbelievers), we found extremely strong evidence for pluralistic ignorance across all 11 countries, from −7.5% (90% CrI = [−10.1%, −5.0%], BF_-+_ → ∞) in Indonesia and up to −20.8% (90% CrI = [−23.4%, −18.2%], BF_-+_ → ∞) in Brazil. Importantly, many people underestimated this consensus, ranging from at least 47.6% in Indonesia to 69.6% in Germany (Fig. 2). Supplement F in the Supplemental Material details the pluralistic ignorance estimates for all belief types and countries as well as tests considering sampling uncertainty in the actual belief estimate.

We did not test Hypotheses 2a through 2d (i.e., that personal climate-change beliefs would moderate misperceptions of others’ climate-change beliefs) because there were few skeptics across countries (weighted *n =* 427; 11.7%) and within countries (China: weighted *n* = 15; Indonesia: weighted *n* = 66).

#### Effectiveness of the public-consensus intervention (Aim 2)

The intervention informed climate-change believers about the actual public consensus on climate change in their country. We found extremely strong support in all countries except Indonesia that climate-change believers underestimated the prevalence of proclimate views (not preregistered; range: −14.5%, 90% CrI = [−16.8%, −12.2%], BF_-+_ → ∞ in Brazil; 2.0%, 90% CrI = [−0.2%, 4.1%], BF_-+_ = 15.16 in Indonesia; Supplement F)—a prerequisite for the intervention to be effective.

**Fig. 3.**
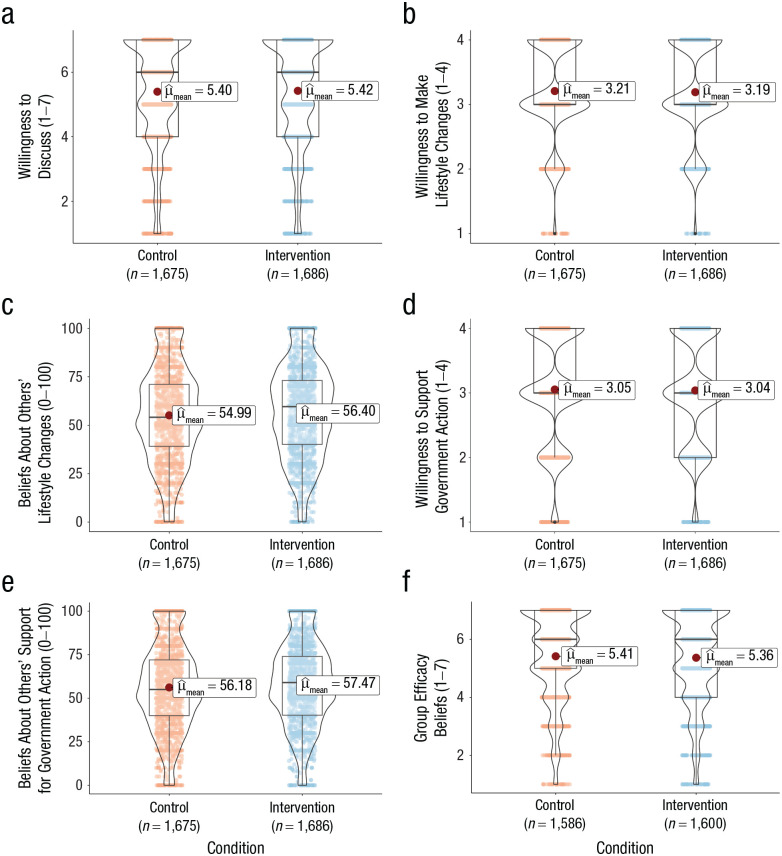
Comparison of the control and intervention condition on the six outcomes: willingness to discuss climate change (a), willingness to make lifestyle changes to mitigate climate change (b), beliefs about others’ willingness to make lifestyle changes to mitigate climate change (c), support for government action on climate change (d), beliefs about others’ support for government action on climate change (e), and group efficacy beliefs (f). The gray line represents the median.

Consistent with the descriptive results (Fig. 3) and the supporting Hypothesis 3, climate-change believers were more willing to express their opinion on climate change after being exposed to the intervention compared with the control message, with a small effect size of Cohen’s δ* =* 0.05 (90% CrI = [−0.02, 0.11], BF_-+_ = 6.15) and little variation across countries (τ_c_ = 0.04, 95% CrI = [0.00, 0.13]; Supplement G). Contrary to Hypothesis 4a, we found extremely strong evidence that the intervention did not change personal willingness to change one’s lifestyle compared with the control message (BF_01_ = 218.35), with little variation across countries (τ_c_ = 0.06, 95% CrI = [0.00, 0.19]). If the effect was present, it would likely be close to zero (*b* = −0.01, 95% CrI = [−0.10, 0.08]). Similarly, expectations about others’ willingness to make lifestyle changes were not significantly higher in the intervention compared with the control condition (Hypothesis 4b, *b =* 1.49%, *t*(3,360) = 1.77, *p* = .077). Contrary to Hypothesis 5a, we found extremely strong evidence that the intervention did not influence personal support for government action (BF_01_ = 232.28), again with little cross-country variation (τ_c_ = 0.05, 95% CrI = [0.00, 0.17]). If the effect was present, it would likely be negligible (*b =* −0.01, 95% CrI = [−0.09, 0.08]). Expectations about others’ government support were also not significantly higher in the intervention compared with the control condition (Hypothesis 5b, *b* = 1.39%, *t*(3,360) = 1.69, *p* = .092). Regarding Research Question 2 (*n* = 3,186), we found extremely strong evidence against any effect on climate-change believers’ efficacy beliefs about whether their country’s citizens could jointly prevent the negative consequences of climate change (BF_01_ = 218.81, *b* = −0.02, 95% CrI = [−0.10, 0.07]), with little variation in the intervention effect across countries (τ_c_ = 0.05, 95% CrI = [0.00, 0.16]).

We refrained from testing whether the intervention was more effective for people with higher rather than lower national identification (Hypotheses 6a and b and Research Question 3) because of very high levels of national identification (Table 2). Even with such high national identification—theoretically providing an ideal basis for the public-consensus message to be effective—the intervention was largely ineffective. Sensitivity and exploratory analyses may be found in Supplement H.

#### Updated perceptions of the public consensus (manipulation check)

The null findings for most of the intervention outcomes may be explained in at least two ways: (a) Participants updated their perceptions of the public consensus without affecting most other outcomes, or (b) they did not update their consensus perceptions, preventing any cascading effects on most outcomes. To test these alternative explanations, we conducted a small follow-up study using quota-based samples in Brazil (*n* = 219 after exclusions following the same criteria as in the main study). This study included a clear manipulation check that assessed updated perceptions of the public consensus—“What percentage of Brazilians, do you believe, would think the following ways about climate change? Please indicate a number from 0% (no one) to 100% (everyone).” See Supplement I for details on the sample, method, and data analysis. We selected Brazil because of the strong public consensus concerning climate change (88%) and the substantial misperceptions (−20.8%; Fig. 2). At the descriptive level, consensus perceptions among climate-change believers were higher after viewing the intervention (*M* = 73.0%, *SD* = 24.8%) compared with the control message (*M* = 63.8%, *SD* = 28.8%). Controlling for prior perceptions of the public consensus, we found that consensus perceptions were 10.8 percentage point higher after viewing the intervention compared with the control message (*t*(218) = 2.58, *p* = .011).

### Discussion

Conceptually replicating Leviston et al.’s (2013) work from Australia, we found broad generalizability of pluralistic ignorance; specifically, people across countries underestimated the prevalence of proclimate views by at least 7.5% (90% CrI = [5.0, 10.1]) in Indonesia and up to 20.8% (90% CrI = [18.2, 23.4]) in Brazil. Contrary to expectations, the public-consensus intervention was largely ineffective, except for a slight increase in willingness to express one’s proclimate opinion (δ = 0.05, 90% CrI = [−0.02, 0.11]). The null results for most intervention outcomes may be explained by (a) a lack of updating consensus perceptions, (b) the use of real-world data as part of the intervention (as opposed to experimentally manipulated data which had been used in the past), and (c) the gap between the outcomes and the public consensus emphasized in the intervention (for more details, see the General Discussion).

A remaining unknown is whether climate-change-related pluralistic ignorance differs across cultural contexts. Tentatively supporting theoretical predictions, Study 1 descriptively shows more pronounced pluralistic ignorance about climate-change beliefs in looser cultures compared with tighter cultures (Fig. 2). In Study 2, we used secondary data from 55 countries to explore these predictions (Table 1).

## Study 2

### Materials and method

#### Description of secondary data sets

We combined country-level summary data from two cross-sectional, secondary data sets: (a) cultural tightness-looseness scores based on student and general population samples across 55 countries (*n* = 22,496; Eriksson et al., 2021) and (b) data used to calculate pluralistic-ignorance scores from the same 55 countries (*n* = 60,230), selected from the Global Climate Change Survey (Andre et al., 2024) and including probability-based, nationally representative samples from 125 countries collected in the Gallup World Poll 2021/2022. Because we worked with anonymized, secondary data sets, this study did not require ethical approval.

#### Actual willingness to fight climate change

The Global Climate Change Survey assessed actual willingness to fight climate change using one item: “Would you be willing to contribute 1% of your household income every month to fight global warming? This would mean that you would contribute [$1] for every [$100] of this income.” Response options included *yes*, *no*, *don’t know*, and *refuse*. Country-level proportions of participants who indicated *yes* had been weighted using the Gallup sampling weights accounting for unequal selection probabilities, nonresponses, and demographics (i.e., at least age and gender).

#### Perceived willingness of others to contribute to fighting climate change

The Global Climate Change Survey assessed perceptions of willingness to fight climate change using one item: “We are asking these questions to 100 other respondents in [country]. How many, do you think, are willing to contribute at least 1% of their household income every month to fight global warming?” Response options ranged from 0 to 100, with additional *don’t know* and *refuse* options.

#### Pluralistic ignorance (outcome)

Country-level pluralistic ignorance was calculated as an accuracy score (Sargent & Newman, 2021)—the difference between the country-level perceptions of others’ willingness and the actual proportion of participants per country who indicated they were willing to contribute financially to fight climate change. Negative values indicate underestimation.

#### Cultural tightness-looseness (predictor)

Cultural tightness-looseness was assessed using the tightness scale that includes six items, such as “There are very clear expectations for how people should act in most situations” and “People in this country almost always comply with social norms.” The measure shows high internal consistency (Cronbach’s α = .80), measurement invariance across countries, and high predictive validity for various phenomena, such as perceived appropriateness of direct punishment and COVID-19 deaths (Eriksson et al., 2021; Gelfand et al., 2011, 2021).

### Data analysis

All analyses were conducted in R (Version 4.1.0; R Core Team, 2021) and *brms* (Version 2.16.3; Bürkner, 2018) using *RStan* (Version 2.21.3; Stan Development Team, 2022). We first fitted two Bayesian linear regression models, with cultural tightness-looseness as a continuous predictor of (a) pluralistic ignorance about willingness to contribute and (b) the actual country-level norm to fight climate change. Both models used weakly informative priors, *b* ~ Normal(0, 10). We then fitted a Bayesian mediation model with cultural tightness-looseness as a predictor, the actual social norm as a mediator, and the perceived social norm as an outcome. Due to the cross-sectional nature of the data, we could not directly test causal relationships; instead, we tested the association of cultural tightness-looseness and perceived norms while accounting for the effect of the actual norm. We used weakly informative priors for the main analysis, *b* ~ Normal(0, 10), and a more informed prior approximated from the posterior of Study 1 (posterior passing; Brand et al., 2019) for the robustness check, *b*_ctl_ ~ Normal(9.34, 4.37). Decision criteria and technical details were identical to those of Study 1.

### Exploratory results: Cultural tightness-looseness as a predictor of pluralistic ignorance (Aim 3)

**Fig. 4.**
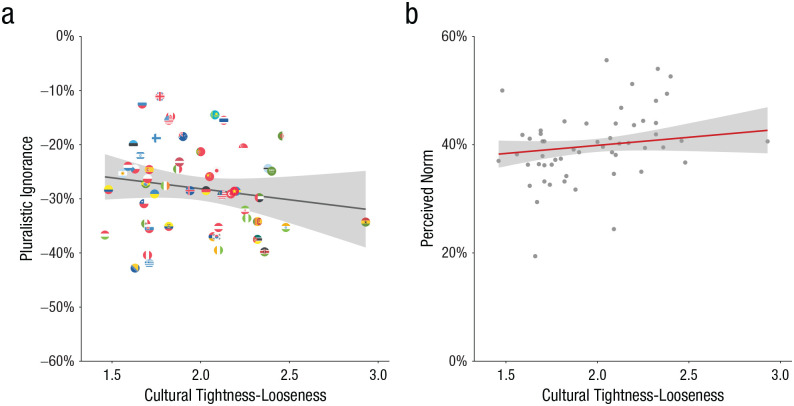
Associations of cultural tightness-looseness with pluralistic ignorance and perceived social norms controlling for the mediating effect of the actual social norm. Lower values indicate more cultural looseness; higher values indicate more cultural tightness. The shaded area represents the 95% confidence interval (CI). In (a) is shown the association between cultural tightness-looseness (*x*-axis) and pluralistic ignorance (*y*-axis) as well as descriptive pluralistic ignorance effects. In (b) is shown the association between cultural tightness-looseness (*x*-axis) and the country-level perceptions of the social norm (*y*-axis), controlling for the actual country-level norm.

Across the 55 countries, people underestimated other people’s willingness to contribute financially to fight climate change in their country, with a mean level of pluralistic ignorance of −28.0%, ranging from −42.8% in Bosnia and Herzegovina to −11.1% in the United Kingdom (see Andre et al., 2024). Contrary to theoretical predictions (Research Question 4), exploratory analyses suggested moderate support for more pronounced pluralistic ignorance in tighter compared with looser cultures (see Figure 4a), BF_-+_ = 6.06, *b* = −3.62, 90% CrI = [−9.14, 1.88]. One potential reason is that tighter rather than looser cultures were more willing to contribute to fighting climate change, BF_-+_ = 102.99, *b* = 11.72, 90% CrI = [3.64, 19.79], and thus had more “room” to underestimate others’ willingness. To account for this possibility, we used a Bayesian mediation model with cultural tightness-looseness as a predictor, actual norms as a mediator, and perceived norms as an outcome. We found strong support that looser compared with tighter cultures perceived weaker norms about willingness to contribute and thus showed more pluralistic ignorance (see Figure 4b), BF_-+_ = 11.21, *b =* 2.99, 90% CrI = [−0.55, 6.54]. A one-step increase in looseness—which corresponds, for example, to the difference between Algeria and Hungary—was associated with around 3 percentage point more pluralistic ignorance. However, this estimate is rather uncertain, given the posterior distribution spans a wide range of values. Robustness checks with more informed priors based on Study 1 yield highly similar results, BF_-+_ = 10.96, *b* = 2.98, 90% CrI = [−0.58, 6.53].

### Discussion

In Study 2, pluralistic ignorance about willingness to fight climate change was more pronounced in looser compared with tighter cultures when controlling for the actual social norm. Although a 3-percentage-point difference is nominally small, it is rather remarkable when considering that more specific factors than cultural tightness-looseness usually produce even smaller shifts in norm perceptions. For example, learning that one’s U.S. state passed a 100% renewable energy mandate shifted norm perceptions about public support for this mandate by 2.5 percentage points (Syropoulos et al., 2024).

## General Discussion

Conceptually replicating Leviston et al.’s (2013) work, we found that people across 11 countries (*n* = 3,653) underestimated the prevalence of proclimate views and overestimated the prevalence of skeptical views (see Table 4), ranging from at least 7.5% (90% CrI = [5.0, 10.1]) in Indonesia up to 20.8% (90% CrI = [18.2, 23.4]) in Brazil. Although the original study showed that Australians underestimated the prevalence of views that climate change is not human-caused (Leviston et al., 2013)—a popular opinion in Australia at that time—we hypothesized and demonstrated the reverse: People across all 11 countries substantially overestimated the prevalence of this current minority opinion. These diverging results were expected because they align with the phenomenon of pluralistic ignorance. Alongside the scientific importance, this work highlights the significance of adapting hypotheses when the context changes. Thus, results of a replication study that are inconsistent with the original finding but consistent with the underlying phenomenon can still constitute a successful replication.

**Table 4.** Summary of the Results Across the Two Studies

Research question or hypothesis	Hypothesis supported?	Summary
**Aim 1: Pluralistic ignorance (Study 1)**		
Hypothesis 1: Pluralistic ignorance of climate-change beliefs	Yes	We found extremely strong evidence for pluralistic ignorance across all 11 countries, from −7.5% in Indonesia (90% CrI = [−10.1%, −5.0%]) and up to −20.8% in Brazil (90% CrI = [−23.4%, −18.2%]).
Research Question 1: Cross-country generalizability of pluralistic ignorance	Yes	Pluralistic ignorance of climate-change beliefs generalized across the 11 studied countries in terms of presence but not magnitude.
Hypothesis 2: Personal climate-change beliefs moderate (mis)perceptions of others’ climate-change beliefs	Not tested	We could not test this hypothesis because there were few climate skeptics across and within countries.
**Aim 2: Effects of the public-consensus intervention (Study 1)**		
Hypothesis 3: Willingness to express one’s proclimate opinion	Yes	We found moderate evidence that people are more willing to express their proclimate opinions in response to the intervention compared with the control message (δ = 0.05, 90% CrI = [−0.02, 0.11]).
Hypothesis 4a: Willingness to make lifestyle changes to mitigate climate change	No	We found extremely strong evidence that the intervention did not change personal willingness to change one’s lifestyle compared with the control message.
Hypothesis 4b: Expectations about fellow citizens’ willingness to make at least some of these lifestyle changes	No	Expectations about others’ willingness to make lifestyle changes were not significantly^1^ higher in the intervention compared with the control condition.
Hypothesis 5a: Prioritization of government action on climate change	No	We found extremely strong evidence that the intervention did not influence the prioritization of government action on climate change.
Hypothesis 5b: Expectations about fellow citizens’ prioritization of government action	No	Expectations about others’ prioritization of government action were not significantly^1^ higher in the intervention compared with the control condition.
Research Question 2: Group efficacy beliefs	No	We found extremely strong evidence against any effect on climate-change believers’ efficacy beliefs about whether their country’s citizens could jointly prevent the negative consequences of climate change.
Hypothesis 6/Research Question 3: National identification	Not tested	We could not test this hypothesis and research question because of very high mean national identification and low variability.
**Study 2: Country-level predictor of pluralistic ignorance (Aim 3)**		
Exploratory Research Question 4: Cultural tightness-looseness	Yes	We found strong exploratory support that looser compared with tighter cultures perceive the norm regarding willingness to contribute to be lower and thus less accurate when controlling for the actual public norm (*b* = 2.99, 90% CrI = [−0.55, 6.54]).

Note: 90% CrI = 90% credible intervals. ^1^To test Hypothesis 4b and Hypothesis 5b, we used frequentist analyses; for all other hypotheses and research questions, we used Bayesian analyses.

Providing actual country-specific information about the public consensus on climate change slightly increased climate-change believers’ willingness to express their opinions on the topic (see Table 4). This has practical implications for climate-change communication: Communicating that most people in a country believe human activity is responsible for climate change may boost discussions around the topic, which breaks the spiral of silence and further reduces pluralistic ignorance (Geiger & Swim, 2016; Noelle-Neumann, 1993). Although the effect is nominally small (δ* =* 0.05, 90% CrI = [−0.02, 0.11]), it may still be of practical importance (Anvari et al., 2022) because of its self-amplifying nature and the scalability of the intervention.

The public-consensus intervention, however, did not robustly influence any of the other outcomes (see Table 4). One potential explanation is that the current public-consensus message may not have increased consensus perceptions, preventing cascading effects on most other outcomes. Although a small follow-up study in Brazil indicated that consensus perceptions were higher in the intervention compared with the control condition, we recognize that this may not hold true for other studied countries, especially given different “dosage” effects (i.e., varying actual consensuses). However, if there was no belief updating in response to the intervention, we would expect a null effect on willingness to express one’s proclimate opinion, contrary to the findings of Study 1. Another explanation may, therefore, be the use of real-world data as part of the public-consensus messages. Past work often relied on nonpolled, more optimistic data, potentially inflating message effectiveness. Supporting this argument, messages emphasizing a higher rather than a lower scientific consensus increased consensus perceptions more (Orchinik et al., 2024).

In addition, the gap between the outcomes and the public consensus emphasized in the message may have played a role. Although consensus messages can influence nontargeted perceptions (Cialdini et al., 1990; van der Linden et al., 2019; Većkalov et al., 2024), the current message neither moved normative expectations nor did it subsequently influence personal willingness to change one’s lifestyle or support for government action. These findings are consistent with a recent 63-country intervention tournament in which a public-consensus message on climate change as a global emergency did not affect any nontargeted climate-mitigation outcomes (Vlasceanu et al., 2024). To spur climate action, it may, therefore, be more expedient to use messages about climate action rather than climate-change beliefs. However, as climate action is not prevalent across contexts (Leiserowitz et al., 2021), such alternative messages may backfire in some countries.

Across 55 countries in Study 2, pluralistic ignorance was more pronounced in looser rather than tighter cultures, once actual social norms were controlled for (see Table 4). Although this finding is exploratory, does not imply causality, and requires further replication, it emphasizes that pluralistic ignorance is not only a phenomenon of numerical cognition but—at least to some extent—associated with broader sociocontextual factors, such as cultural differences.

Despite these advancements, we recognize several limitations that present fruitful avenues for future work. First, given the limited number of countries in Study 1, future research should explore whether public-consensus interventions are differentially effective in tighter compared with looser cultures (see also Bergquist, 2025). Cultural tightness-looseness theory may predict that such interventions are more effective in tighter cultures, because such cultures have higher feelings of accountability and less tolerance for norm violations (Gelfand et al., 2011). In contrast, misperception-correction approaches (Lees & Cikara, 2020) would predict that consensus interventions may be more effective in looser cultures, where people may be less accurate.

Second, we used cross-quota samples (age and sex) in Study 1, as is common in international surveys (e.g., Gallup World Poll; https://news.gallup.com/poll/165404/world-poll-methodology.aspx), because of the limited availability of census data in some countries. Given that the samples are not fully representative, we would need to be cautious about generalizing these findings beyond the more urban and highly educated parts of the studied populations. However, previous research has suggested that demographics (e.g., education, gender) do not predict misperceptions of voters’ support for several environmental policies; rather, one of the most important predictors is one’s support for the policies (Lees et al., 2023). This may imply that one’s climate-change beliefs, rather than demographic characteristics, are most likely to predict pluralistic ignorance. In the absence of fully representative samples, therefore, we reweighted the existing cross-quota samples to match the national parameters of climate-change beliefs to draw more valid conclusions about pluralistic-ignorance effects.

Third, the intervention design in Study 1 was limited by preexisting cross-country data. This allowed for testing the effectiveness of an emerging, real-world intervention—communicating how many members of the public believe climate change is human-caused. However, this intervention may not necessarily be the most effective approach, as it may be too far removed from the outcomes. This calls for quantifying and updating different types of public consensus in a way that is consistent across countries. Richer secondary data about emerging consensuses would allow for selecting the most promising intervention.

Last, the secondary data in Study 2 allowed only for testing country-level but not region-level cultural tightness-looseness as a predictor of pluralistic ignorance. Given that cultural tightness-looseness varies, especially within larger countries (e.g., China and the United States; Gelfand, 2019; Harrington & Gelfand, 2014), future research may account for within-country regional tightness-looseness (Chen et al., 2022) to potentially improve predictions of pluralistic ignorance.

In conclusion, people across 11 countries substantially underestimated others’ proclimate views. Such tendencies seemed to be more pronounced in looser cultures. Although pluralistic ignorance was widespread, raising awareness about the broad public consensus on human-caused climate change did not seem to promote climate action, except for individuals’ willingness to express their proclimate opinions.

## Supplemental Material

sj-docx-1-pss-10.1177_09567976251335585 – Supplemental material for What We Think Others Think and Do About Climate Change: A Multicountry Test of Pluralistic Ignorance and Public-Consensus Messaging Supplemental material, sj-docx-1-pss-10.1177_09567976251335585 for What We Think Others Think and Do About Climate Change: A Multicountry Test of Pluralistic Ignorance and Public-Consensus Messaging by Sandra J. Geiger, Jana K. Köhler, Zenith N. C. Delabrida, Karla A. Garduño-Realivazquez, Christian A. P. Haugestad, Hirotaka Imada, Aishwarya Iyer, Carya Maharja, Daniel C. Mann, Michalina Marczak, Olivia Melville, Sari R. R. Nijssen, Nattavudh Powdthavee, Radisti A. Praptiwi, Gargi Ranade, Claudio D. Rosa, Valeria Vitale, Małgorzata Winkowska, Lei Zhang and Mathew P. White in Psychological Science

## Footnotes

1. Sparkman et al.’s article became available after the current study was preregistered, and Andre et al.’s article was published after conducting the current work and writing this article. We mention these findings for completeness in the introductory portion of the article, but they did not contribute to the original reasoning behind the tested hypotheses.

2. The minimum time for careful completion was 5 min during pilot testing with participants recruited from our personal networks. Because participants on data-collection panels regularly take part in surveys and are thus often faster than average people, we opted for 3 min as a slightly less conservative threshold for speeding.

3. For exploratory purposes, we also assessed personal beliefs about biodiversity loss and perceptions of others’ beliefs about biodiversity loss. The order of the climate-change and biodiversity-loss blocks was counterbalanced, so that half of the participants per country first completed the block about climate change, but the other half first completed the block about biodiversity loss. The results are beyond the scope of the current article.

## Transparency

*Action Editor:* Jennifer Gutsell

*Editor:* Simine Vazire

*Author Contributions*

**Sandra J. Geiger:** Conceptualization, Data curation, Formal analysis, Funding acquisition, Investigation, Methodology, Project administration, Resources, Software, Visualization, Writing – original draft, Writing – review & editing.

**Jana K. Köhler:** Conceptualization, Funding acquisition, Methodology, Writing – review & editing.

**Zenith N. C. Delabrida:** Methodology, Writing – review & editing

**Karla A. Garduño-Realivazquez:** Methodology, Writing – review & editing.

**Christian A. P. Haugestad:** Methodology, Writing – review & editing.

**Hirotaka Imada:** Methodology, Writing – review & editing.

**Aishwarya Iyer:** Methodology, Writing – review & editing.

**Carya Maharja:** Methodology, Writing – review & editing.

**Daniel C. Mann:** Validation.

**Michalina Marczak:** Methodology, Writing – review & editing.

**Olivia Melville:** Methodology, Writing – review & editing.

**Sari R. R. Nijssen:** Conceptualization, Funding acquisition, Writing – review & editing.

**Nattavudh Powdthavee:** Methodology, Writing – review & editing.

**Radisti A. Praptiwi:** Methodology, Writing – review & editing.

**Gargi Ranade:** Methodology, Writing – review & editing.

**Claudio D. Rosa:** Methodology, Writing – review & editing.

**Valeria Vitale:** Methodology, Writing – review & editing.

**Małgorzata Winkowska:** Methodology, Writing – review & editing.

**Lei Zhang:** Methodology, Writing – review & editing.

**Mathew P. White:** Conceptualization, Funding acquisition, Supervision, Writing – review & editing.

**Artificial intelligence:** No artificial-intelligence-assisted technologies were used in this research or the creation of this article.

**Ethics:** This research complies with the Declaration of Helsinki (2023), and Study 1 received approval from the ethics board at the University of Vienna (Project No. 00769 and Project No. 00843).

**Open Practices:** Study 1 disclosures. Preregistration: The research aims/hypotheses, methods, and analysis plan were preregistered on PsychArchives (June 29, 2022; https://doi.org/10.23668/psycharchives.7059) prior to data collection on September 14, 2022. There were major and minor deviations from the preregistration (for details, see Supplement B in the Supplemental Material). Materials: All study materials are publicly available (https://doi.org/10.17605/OSF.IO/6W5BD). Data: All primary data are publicly available (https://doi.org/10.17605/OSF.IO/R2BYZ). Analysis scripts: All analysis scripts are publicly available (https://doi.org/10.17605/OSF.IO/GWEZM). Computational reproducibility: The computational reproducibility of the results has been independently confirmed by the journal’s STAR team. Study 2 disclosures. Preregistration: No aspects of the study were preregistered because we used secondary data and had to access the data to decide whether they could be used for testing the proposed hypothesis. Materials: All study materials are publicly available (https://doi.org/10.17605/OSF.IO/N62AD). Data: Primary data is not reshared, but all primary data are publicly available via Andre et al. (2024; https://doi.org/10.15185/gccs.1) and Eriksson et al. (2021; https://osf.io/pm5kc/). The combined secondary data set is publicly available (https://doi.org/10.17605/OSF.IO/N73AE). Analysis scripts: All analysis scripts are publicly available (https://doi.org/10.17605/OSF.IO/9H756). Computational reproducibility: The computational reproducibility of the results has been independently confirmed by the journal’s STAR team.
