# Supplementary material for: What We Think Others Think and Do About Climate Change: A Multicountry Test of Pluralistic Ignorance and Public-Consensus Messaging
Source: Psychol Sci. 2025 May 22;36(6):421–42. doi: 10.1177/09567976251335585 (PMC13428961; doi:10.1177/09567976251335585)
Supplement: sj-docx-1-pss-10.1177_09567976251335585 – Supplemental material for What We Think Others Think and Do About Climate Change: A Multicountry Test of Pluralistic Ignorance and Public-Consensus Messaging [file sj-docx-1-pss-10.1177_09567976251335585.docx]

**Supplement A**

**Comparison of the Original Study and the Present Conceptual Replication (Study 1)**

| **Comparison Dimension** | **Original Study** | **Present Replication Study** | | **Reason for Change** | |  |
| --- | --- | --- | --- | --- | --- | --- |
| **Hypotheses** | The original study found that the number of attribution skeptics (i.e., climate change is happening but not human-caused) is underestimated (perceived: 23.7% *vs*. actual: 40.2% in 2010). | Individuals overestimate the number of (c) attribution and (d) trend skeptics in their country. | | We expected that the number of attribution skeptics would be overestimated because descriptive evidence shows that attribution skeptics are a minority of 5% (Brazil, China, and Japan) to 18% (Indonesia) in all countries of interest^1^. This updated hypothesis is consistent with findings from later US studies on misperceptions of climate change beliefs^5^. | |  |
| **Sample** |  |  | |  | |  |
| Sample nature | Nationally representative sample of Australians (T1: 48.8% women; > 24-85+ years; 86% in urban area) | Cross-quota sample based on age and sex for each of the 11 countries (50.4% women, 18-85 years with *M* = 43.7; 77.3% in urban areas) | |  | |  |
| Sample size | *n* = 5,036  two waves | *n* = 3,653  one wave | | We used a smaller sample size to make the data collection feasible across as many countries as possible. This sample size, nevertheless, allowed us to reliably detect very small pluralistic ignorance effects of 5%. | |  |
| Recruitment | Accredited online panel provider | Accredited online panel providers | |  | |  |
| Mode of data collection | Online | Online | |  | |  |
| **Design** |  |  | |  | |  |
| Own climate change beliefs | Which of the following statements best describes your thoughts on climate change?   - I don’t think that climate change is happening. - I have no idea whether climate change is happening or not. - I think that climate change is happening, but it’s just a natural fluctuation in Earth’s temperatures. - I think that climate change is happening, and I think that humans are largely causing it.   The display order of the options was fixed, as shown above. | In general, which of the following statements, if any, best describes your view?   - The climate is changing, and human activity is mainly responsible. - The climate is changing, and human activity is partly responsible, together with other factors. - The climate is changing but human activity is not responsible at all. - The climate is not changing. - I don’t know.   The display order of the options was counterbalanced across participants:   1. Mainly human-caused Partly human-caused Not human-caused No climate change Don’t know 2. No climate change Not human-caused Partly human-caused Mainly human-caused Don’t know | | We used a measure previously employed in the multi-country YouGov Globalism survey, with five rather than four response options. We used this measure to (a) capture widely held beliefs that climate change is caused by both natural processes and human activity (27-64% depending on the country (YouGov Cambridge, 2020)), (b) test a public consensus intervention—which uses pre-existing, real-world data on the actual distribution of climate change beliefs in each of the studied countries, and (c) to compare the perceived beliefs of others against the actual beliefs from the large-scale YouGov survey as a robustness check. The content of our categories closely matches the content of the four original categories (see color coding of the items), though the wording differs. We believe that the fifth category is important since climate change beliefs have become more nuanced (not just natural *vs*. human-caused), as is indicated by the YouGov data, where this belief in partly natural, partly human-caused climate change is the most popular or second most popular belief in each of the 11 studied countries.  We also decided to vary the response order in which the response options are displayed since we do not know whether and how the order of response options affects second-order beliefs. | |  |
|  |  | |  | |  | |
| Perceptions of others’ climate change beliefs | Try and guess the percentage of Australians who would think the following ways about climate change (HINT: the numbers you place beside all four boxes should add up to 100). The survey logic was set up in a way that participants could only proceed if the numbers added up to 100%.  The display order of the options was fixed (see personal climate change beliefs). | | What percentage of [country citizens] do you believe would think the following ways about climate change? Please indicate a number from 0% (no one) to 100% (everyone) for the following statements such that they sum up to 100%.”  The display order of the options was counterbalanced across participants (see personal climate change beliefs). | | We adapted the wording to   1. reflect that we are interested in participants’ beliefs rather than implying that there is a correct answer, and their guess should get as close as possible (“try and guess”) to this correct answer. 2. help participants understand better what 0% and 100% imply. 3. match the slider scale we used. | |
| Attention check and bot detection | No information. | | With this question, we would like to ensure that participants pay attention. Please select the option ‘Red’ from the list below.  *Blue, Red, Yellow, Green,* and *White* | | To ensure high data quality, we added an attention check and reCAPTCHA bot detection. We added the attention check at the end of the survey, before the demographics, to avoid the check influencing the results. | |
| Additions | Second wave to test the stability of beliefs | | Intervention to causally test whether informing people about the actual beliefs in their country affects factors related to climate action. | | Since disclosing the actual beliefs on climate change is increasingly used as an intervention in the real world, we believe that testing what outcomes the intervention can affect is more valuable than replicating the stability of beliefs. | |

**Supplement B**

**Deviations from the Preregistration**

| **Section** | **Preregistered** | **Deviation and Reason** |
| --- | --- | --- |
| **Introduction:**  **Hypotheses numeration** | Effects of the intervention on:  H3a: expectations about others’ willingness to make lifestyle changes  H3b: personal willingness to make lifestyle changes  H4a: expectations about others’ support for government action  H4b: personal support for government action  H5: willingness to discuss climate change  H6c: moderation by national identification on group efficacy beliefs | We changed the numeration of the hypotheses as follows:  H3: willingness to discuss climate change (previously H5)  H4a: personal willingness to make lifestyle changes (previously H3b)  H4b: expectations about others’ willingness to make lifestyle changes (previously H3a)  H5a: personal support for government action (previously H4b)  H5b: expectations about others’ support for government action (previously H4a)  RQ3: moderation by national identification on group efficacy beliefs (previously H6c; since the main effect of the intervention on group efficacy is a research question as well (RQ2), we adapted H6c to RQ3). |
| **Methods:**  **Power simulations** | We ran a priori power analyses using country-level one-sample *t*-tests and country-level regression analyses. | As we planned to use frequentist quasibinomial regressions for the final analyses (instead of *t*-tests in the preregistration) to better model the 0-100% data, we reran the power simulations with quasibinomial regressions. |
| **Methods: Sample size** | We aim for 330 participants per country. | As stated in the note of Table 2, in some countries, the sample size deviates from the preregistered *n*= 330 because (a) a small number of potential bots were excluded (Canada, Germany, Italy, Japan, Mexico), (b) several participants completed the survey at the same time while the quota had just been reached (Brazil, China, Thailand), or the panel provider recruited an additional 10% due to potential bots (Japan). |
| **Methods: Attention check** | We preregistered that we would check participants’ attention with one item before participants are presented with the control/intervention message: “Please select the option ‘neutral’ and proceed to the following question.” with a response scale from 1 *strongly disagree*, 3 *neutral*, and 5 *strongly agree*. | We decided to include the attention check at the end of the survey so as not to influence participants’ responses. Based on the panel provider’s request, we changed the attention check to: “With this question, we would like to ensure that participants pay attention. Please select the option ‘Red’ from the list below.” The list included the options *Blue*, *Red*, *Yellow*, *Green*, and *White*. |
| **Methods: Comprehension check** | We preregistered to check participants’ comprehension with the following item: “Comprehension will be checked using one item at the end of the survey: Which of these messages have you seen previously in this survey?” | Based on the panel provider’s request, we needed to drop the comprehension check, as it was deemed too difficult for participants. Thus, we also dropped the inclusion criteria of ‘passed comprehension check.’ |
| **Methods: Items** | We preregistered that we would assess discussing biodiversity loss using one item: “How often do you discuss biodiversity loss with others?” Response options are *often*, *occasionally*, *rarely*, and *never*. | As some participants took longer than 10 minutes during the pretesting, we decided to drop some of the items from the survey, including the willingness to express one’s opinion on biodiversity loss. Therefore, the randomization (preregistered in Table 4) was not applicable anymore, and we had four instead of eight randomizations: (1) CC own, CC other, BL own, BL other; (2) CC other, CC own, BL other, BL own; (3) BL own, BL other, CC own, CC other; and (4) BL other, BL own, CC other, CC own. |
| **Methods: Control and intervention message** | We had preregistered the following control message:  “Previously, you estimated that [x] out of 100 [NATIONALITY] believe that the climate is changing, and human activity is partly ([x]) or mainly ([x]) responsible.” | We changed “[x] out of 100” to “[x]%” as participants in the pretest deemed this more accurate and comprehensible.  The same changes were applied to the intervention message. |
| **Methods:**  **Own willingness to make lifestyle changes and support for government action** | We had preregistered the following wording of the two outcomes:  “Consider what [NATIONALITY] believe about climate change. How much, if anything, would you be willing to change about how you live and work to help reduce the effects of climate change?” and  “Consider what [NATIONALITY] believe about climate change. Do you think climate change should be a very high, high, medium, or low priority for the government of [COUNTRY]?” | As participants during the pretesting were confused about considering others’ opinions for this question, we rephrased the items as follows:  “How much, if anything, would you be willing to change about how you live and work to help reduce the effects of climate change?”  “Do you think climate change should be a very high, high, medium, or low priority for the government of [country]?” |
| **Methods:**  **Group efficacy beliefs** | We had preregistered to use three items as per van Zomeren et al. (2010). | We selected one of the three items, based on participants’ feedback during the pretesting, that the items were redundant and thus confusing, which was also confirmed by the high reliability (α = 94; van Zomeren et al., 2010).  Consequently, we needed to deviate from the analysis plan to conduct reliability analyses and average across all items. |
| **Methods:**  **Age** | We had preregistered that we would assess age using two items, one to control for in the analyses and one for the quotas:   1. What year were you born? 2. How old are you? 18-29 years, 30-39 years, 40-49 years, 50-59 years, 60 years or older | We assessed age with one item as a control variable:  “How old are you?” Participants could then select their age (in years). For the quota count, we used embedded data that categorized the response into one of five age groups (18-29 years, 30-39 years, 40-49 years, 50-59 years, 60 years or older). |
| **Analysis:**  **Bot exclusions** | ­­— | We added bot detection and excluded responses that likely were provided by bots (reCAPTCHA < .50 as recommended by [Qualtrics](https://www.qualtrics.com/support/de/survey-platform/survey-module/survey-checker/fraud-detection/?rid=langMatch&prevsite=en&newsite=de&geo=AT&geomatch=)) from the analyses. These exclusions are unlikely to change the results, as only 16 responses were excluded. |
| **Analysis: Hypothesis 1a-d** | We had preregistered to conduct Bayesian multi-level Gaussian regressions with participants at level 1 and countries at level 2. | We conducted Bayesian multi-level zero-one-inflated regression analyses with the four outcomes (i.e., the estimated percentage of people who (a) believe in mainly human-caused climate change, (b) believe in partly human-caused climate change, (c) do not believe in human-caused climate change, and (d) do not believe in climate change). We used this strategy instead of the preregistered analysis plan, as posterior predictive checks of both Gaussian and skew-normal models indicated that the actual data are not accurately represented by the model. |
| **Analysis: Hypothesis 1a-d** | — | We calculated sampling weights using automated raking and reweighted the existing cross-quota samples (age and sex) based on the distribution of climate change beliefs in the YouGov survey to ensure adequate representation of all belief groups. |
| **Analysis: Hypothesis 1a-d** | — | To incorporate sampling uncertainty into the actual percentages of beliefs from the YouGov survey, we compared the perceived percentages against a region of practical equivalence (ROPE), defined as the actual percentage from the YouGov survey ± the margins of error based on 95% credible intervals. |
| **Analysis: Hypothesis 2a-d** | We preregistered that we would test Hypothesis 2a-d:  “We, therefore, expect that trend skeptics overestimate the number of trend skeptics more than individuals with other climate change beliefs (i.e., ‘happening but not human-caused’, ‘don’t know’, ‘happening and partly human-caused’, ‘happening and mainly human-caused’; Hypothesis 2a) do.  Attribution skeptics will overestimate the number of attribution skeptics more than individuals with other climate change beliefs (i.e., ‘not happening’, ‘don’t know’, ‘happening and partly human-caused’, ‘happening and mainly human-caused’; Hypothesis 2b) do.  Those who believe that climate change is partly human-caused will underestimate the size of this group less than individuals with other climate change beliefs (i.e., ‘not happening’, ‘happening but not human-caused’, ‘don’t know’, ‘happening and mainly human-caused’; Hypothesis 2c) do.  Those who believe that climate change is mainly human-caused will underestimate the size of this group less than individuals with other climate change beliefs (i.e., ‘not happening’, ‘happening but not human-caused’, ‘don’t know’, ‘happening and partly human-caused’; Hypothesis 2d) do.” | Due to an unexpectedly low number of climate change non-believers in our samples, we could not test these hypotheses. |
| **Analysis: Hypothesis 6a and b, Research Question 3** | We had preregistered to test whether national identification moderates the effectiveness of the intervention. | We did not test any hypotheses or research questions about national identification due to very high national identification scores and low variance in our samples. |
| **Analysis:**  **Hypothesis testing** | We had preregistered that we would use one-sided hypothesis tests for all outcomes except group efficacy beliefs. | Since descriptive statistics indicated no differences between the intervention and control condition on any of the outcomes except willingness to express one’s opinion on climate change, we used two-sided hypothesis tests for the five outcomes and a one-sided test for willingness to express one’s opinion. |
| **Exploratory Analyses** | We had preregistered that we would conduct exploratory analyses regarding the pluralistic ignorance effect for biodiversity loss. | These analyses will be reported in a separate manuscript. |

**Supplement C**

**Details Regarding the Experimental Manipulation**

**Figure C1**

*Graphical Representation of the Intervention Message (Example: Canada, English version)*


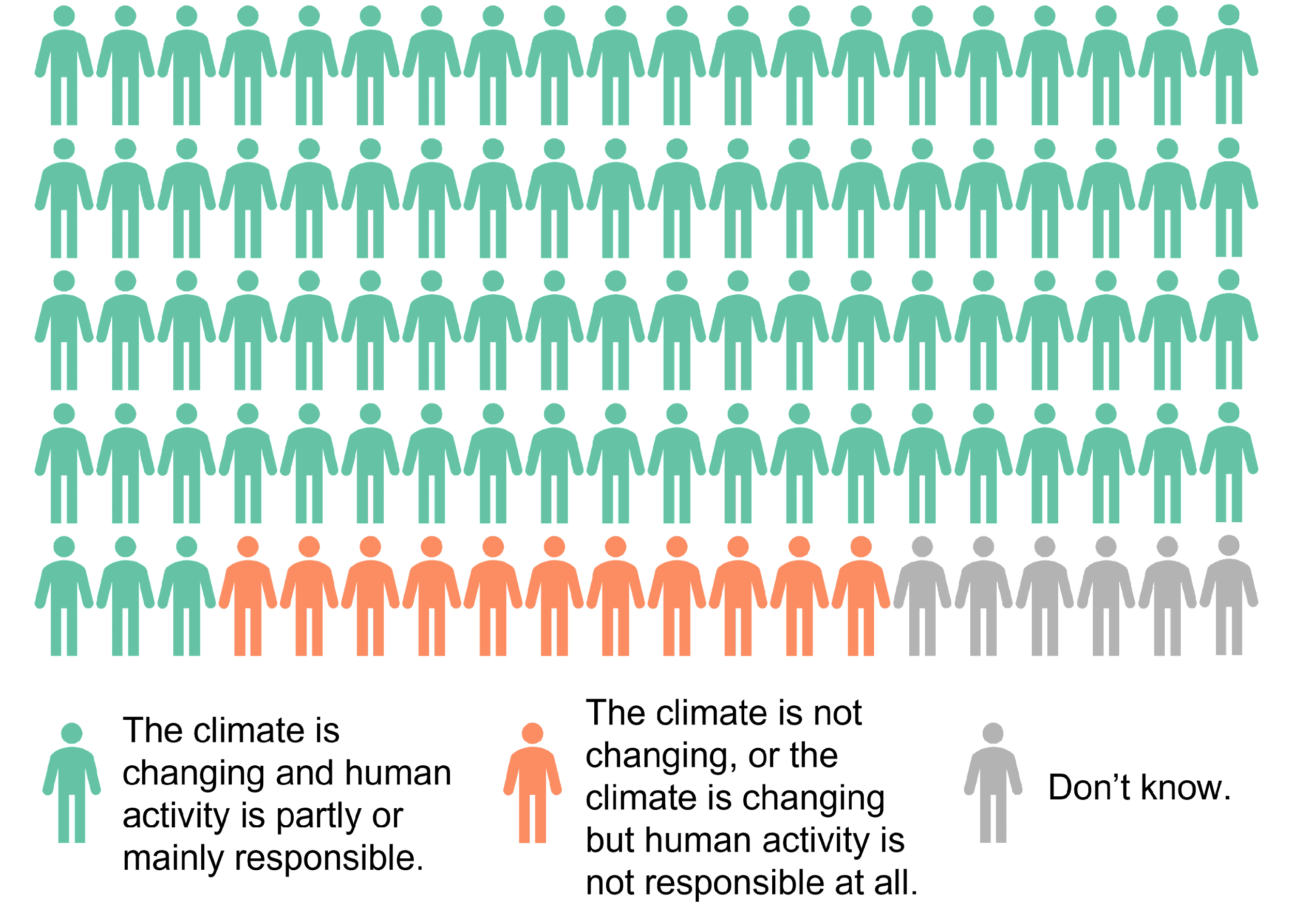


*Note.* Icons created by Uniconlabs - Flaticon, <https://www.flaticon.com/free-icons/person>.

**Table C1**

*Actual Climate Change Beliefs by Country Presented as Part of the Intervention
(Source: YouGov Globalism Survey, 2020)*

| **Country** | **The climate is changing, and human activity is partly or mainly responsible.** | **The climate is not changing, or the climate is changing but human activity is not responsible at all.** | **Don’t know.** |
| --- | --- | --- | --- |
| Brazil | 88% | 9% | 3% |
| Canada | 83% | 11% | 6% |
| China | 87% | 7% | 6% |
| Germany | 83% | 10% | 7% |
| India | 78% | 16% | 6% |
| Indonesia | 71% | 21% | 8% |
| Italy | 86% | 8% | 6% |
| Japan | 85% | 7% | 8% |
| Mexico | 82% | 16% | 2% |
| Poland | 82% | 12% | 6% |
| Thailand | 79% | 15% | 6% |

*Note.* The numbers in the first column, “The climate is changing, and human activity is partly or mainly responsible,” were presented in the intervention message. The intervention graphic was based on the numbers in the first column (green icons), the numbers in the second column (orange icons), and the numbers in the last column (gray icons). If the categories summed up to more than 100% due to rounding, we adjusted the percentage in the “Don’t know” category by ±1%.

**Supplement D**

**Translation and Country-Specific Adaptations of the Translations**

Materials were translated using a standard forward and back-translation approach adapted from the Psychological Science Accelerator (Forscher et al., 2020; Jarke et al., 2022). Materials were first translated from English to one of the local languages by a native speaker and then back-translated by a second, independent native speaker. The back-translation was compared to the original English version, and disagreements were resolved through discussion between the two translators. The final version was proofread by several individuals from the target population in terms of clarity and comprehensibility. Country-specific deviations are listed in Table D1.

**Table D1**

*Country-Specific Adaptations of the Translations*

| **Country** | **Item** | **Adaptation** |
| --- | --- | --- |
| Brazil |  | None. |
| Canada | Own climate change beliefs | “In general, which of the following statements, if any, best describes your view?” is not really feasible in French. Therefore, we removed "if any" from the sentence. |
| China | Own climate change beliefs    Discuss, own changes, own support, efficacy | In **your view** $\to$您个人的想法 (meaning your **individual** thought). We changed this to improve the flow of the sentence, emphasize it is “your” view, and be more consistent with the others’ climate change questions. The original English term (climate change) was added in parentheses.  Do **you** think $\to$ **您个人**认为 (meaning **you, on your own**, think). This is also to emphasize it is “your” view/thought.  For all questions containing “you”, a formal “you” (您) was used in Chinese. |
| Germany |  | For all questions containing “you”, a formal “you” (Sie) was used in German. |
| India | Own and others’ climate change beliefs | “The climate is changing, and human activity is partly responsible, together with other factors.” We used “reasons” instead of “factors” in Hindi. |
| Indonesia |  | None. |
| Italy | Embedded data  Others’ climate change beliefs    Others’ climate change beliefs, expectations change, and government support | A new embedded field (*Nationality_Italian*) that corresponds to *Italian* (singular, just in the masculine form that is the general one used in this type of question) was created since, in Italian; we would say, “*Are you an Italian citizen*?” instead of “Are you a citizen of Italy?”.  To make the instructions clearer, the last sentence (*such that they sum up to 100%*) has been slightly modified as follows: “*in modo tale che Il risultato della loro somma sia 100%”*.  In English, it would be similar to: *such that the result of their sums will be 100%.*  The translated versions of the questions seemed very complicated in Italian and we modified the questions to improve readability: *"Secondo te, quale percentuale di ${e://Field/Nationality_plural} sarebbe ... / pensa che ...".*  *In English, this would be: According to you / In your opinion, What percentage of ${e://Field/Nationality_plural}, would be ... / think ...?* |
| Japan | National identification | In Japanese, there is no equivalent for “to identify”, and we used 認識している (= to recognize) instead in the Japanese version. |
| Mexico | Own lifestyle changes | “How much, if anything, would you be willing to change about how…” As the direct translation was not comprehensible in Spanish, we instead used “How willing would you be to change the way you …” |
| Poland | Own climate change beliefs  Expectations change and government support, efficacy  Expectations change  Efficacy  Political orientation | “In general, which of the following statements, if any, best describes your view?” As using the singular of the word ‘view’ does not work in Polish, we used ‘views’ (plural) instead to keep the same meaning.  We had to add a verb to this sentence: “For the following question” since it would not be feasible if translated directly. The closest translation of the Polish version would be: “Replying for the following question [...]”.  We changed “how they live and work” to a sentence that would be directly translated to “their life and work.” The original was not feasible in Polish.  We have slightly modified one answer category from “very much” to “a very high extent” in Polish because “very much” does not fit here in Polish.  The term “political orientation” (“orientacja polityczna”) is rarely used in spoken language, so we decided to change it into “political views” to make it easier to understand. The meaning is identical. |
| Thailand |  | None. |

**Supplement E**

**Decision Criteria and Technical Details for Study 1 and 2**

**Decision Criteria**

Conclusions regarding the hypotheses and research questions in both studies are based on the posterior distribution, credible intervals, and the Bayes factor, including its standard inference criteria (Table E1). Credible intervals (CrIs) indicate that the true population estimate would fall within this interval with a certain probability (one-sided tests: 90%; two-sided tests: 95%), given the priors and the observed data (Hespanhol et al., 2019). The Bayes factor (BF) quantifies the strength of evidence in favor of a hypothesis over another hypothesis (Hoijtink et al., 2019). In the current paper, BF_10_ and BF_01_ indicate two-sided testing of two competing hypotheses—that the effect differs from zero (H_1_) and that the effect is exactly zero (H_0_). For example, BF_10_ = 10 would indicate that the data are ten times more likely under H_1_ than H_0_, whereas BF_01_ = 10 would mean that the data are ten times more likely under H_0_ than H_1_. In contrast, BF_+-_ and BF_-+_ indicate one-sided testing of two competing hypotheses—that the effect is positive (H_+_) and that the effect is negative (H_-_). Therefore, BF_+-_ = 10 would mean that the data are ten times more likely under H_+_ than H_-_, whereas BF_-+_ = 10 would indicate the opposite.

To draw conclusions about the effects of pluralistic ignorance, we additionally use the region of practical equivalence (ROPE; Kruschke, 2018; Makowski et al., 2019). This allows us to test not only whether the perceived percentages in our samples are *exactly* equal to the actual percentages in the YouGov survey (YouGov Cambridge, 2020) but also whether they are equal to a range of actual percentages (i.e., practically equivalent). We define the ROPE as the actual percentages of climate change beliefs in the YouGov survey ± the sampling uncertainty as indicated by the margins of error based on 95% confidence intervals. These additional analyses have not been preregistered.

**Table E1**

*Standard Inference Criteria for Bayes Factors*

| **BF_10_**  **Evidence for H_1_** | **Interpretation** | **BF_01_**  **Evidence for H_0_** | **Interpretation** |
| --- | --- | --- | --- |
| ≥ 100 | The effect is *extremely* supported by the evidence. | ≥ 100 | The null effect is *extremely* supported by the evidence. |
| 30 ≤ BF_10_ < 100 | The effect is *very strongly* supported by the evidence. | 30 ≤ BF_01_ < 100 | The null effect is *very*  *strongly* supported by the evidence. |
| 10 ≤ BF_10_ < 30 | The effect is *strongly* supported by the evidence. | 10 ≤ BF_01_ < 30 | The null effect is *strongly* supported by the evidence. |
| 3 ≤ BF_10_ < 10 | The effect is *moderately* supported by the evidence. | 3 ≤ BF_01_ < 10 | The null effect is *moderately* supported by the evidence. |
| 1 < BF_10_ < 3 | The evidence is *insufficient* to make a decisive decision, although the effect likely exists. | 1 < BF_01_ < 3 | The evidence is *insufficient* to make a decisive decision, although the null effect likely exists. |
| 1 | No evidence | 1 | No evidence |

*Note.* Adapted from Lieberoth et al. (n.d.) and interpretation based on Jeffreys (1961).

**Technical Details**

All Bayesian models in Studies 1 and 2 were fitted with four chains, each with 20,000 iterations, of which 5,000 per chain served as a warm-up. Posterior convergence is evaluated based on trace plots, $\hat{R}$values, the effective sample size, and divergent transitions. Model fit is assessed based on posterior predictive checks. For all models across both studies, diagnostics were good: the chains converged, the effective sample sizes were large, all $\hat{R}$s were close to 1.0, there were no divergent transitions, and posterior predictive checks showed that the models adequately describe the data.

**Supplement F**

**Pluralistic Ignorance Among the Entire Sample (F1) and Among Change Believers (F2)**

**Table F1**

*Summary of Pluralistic Ignorance Effects Across all Countries*

| **Country** | **Mean pluralistic ignorance [90% CrI]** | **Bayes factor** | **Evidence in favor of / against Hypothesis** | **% inside region of practical equivalence**  **(ROPE)** |
| --- | --- | --- | --- | --- |
| ***H1a:*** *‘mainly human-caused’* | | | | |
| Brazil | -12.3% [‑15.1, ‑9.3] | BF_-+_ → ∞ | Extreme  **●●●●●** | 0.0% |
| Canada | -1.2%  [-3.5, 1.2] | BF_-+_ = 3.78 | Moderate  **●●**○○○ | 92.2% |
| China | 17.3%  [15.2, 19.6] | BF_+-_ → ∞ | Extreme  ●●●●● | 0.0% |
| Germany | 5.4% [3.2%, 7.6%] | BF_+-_ → ∞ | Extreme  ●●●●● | 0.0% |
| India | -8.5%  [-11.1, -5.8] | BF_-+_ → 59,999.00 | Extreme  ●●●●● | 0.0% |
| Indonesia | 3.8% [1.5, 6.2] | BF_+-_ = 311.53 | Extreme  ●●●●● | 20.7% |
| Italy | -2.7%  [-5.1, -0.2] | BF_-+_ = 24.58 | Strong  ●●●○○ | 49.9% |
| Japan | 5.7%  [3.3, 8.1] | BF_+-_ → ∞ | Extreme  ●●●●● | 0.7% |
| Mexico | -8.5%  [-11.1, -5.9] | BF_-+_ → ∞ | Extreme  ●●●●● | 0.0% |
| Poland | -5.5%  [-7.9, -3.1] | BF_-+_ =19,999.00 | Extreme  ●●●●● | 2.0% |
| Thailand | -6.0%  [-8.3, -3.8] | BF_-+_ → ∞ | Extreme  ●●●●● | 0.0% |
|  |  |  |  |  |
| ***H1b:*** *‘partly human-caused’* | | | | |
| Brazil | -7.6%  [-9.5, -5.7] | BF_-+_ → ∞ | Extreme  ●●●●● | 0.0% |
| Canada | -14.1%  [-15.8, -12.3] | BF_-+_ → ∞ | Extreme  ●●●●● | 0.0% |
| China | -29.1% [-31.0, -27.2] | BF_-+_ → ∞ | Extreme  ●●●●● | 0.0% |
| Germany | -21.7% [-23.3, -20.0] | BF_-+_ → ∞ | Extreme  ●●●●● | 0.0% |
| India | -2.9% [-4.7, -1.0] | BF_-+_ = 195.08 | Extreme  ●●●●● | 42.7% |
| Indonesia | -10.3% [-12.1, -8.4] | BF_-+_ → ∞ | Extreme  ●●●●● | 0.0% |
| Italy | -10.6%  [-12.3, -8.9] | BF_-+_ → ∞ | Extreme  ●●●●● | 0.0% |
| Japan | -18.9%  [-20.7, -17.0] | BF_-+_ → ∞ | Extreme  ●●●●● | 0.0% |
| Mexico | -7.6%  [-9.3, -5.8] | BF_-+_ → ∞ | Extreme  ●●●●● | 0.0% |
| Poland | -12.7%  [-14.4, -11.0] | BF_-+_ → ∞ | Extreme  ●●●●● | 0.0% |
| Thailand | -8.7%  [-10.3, -7.0] | BF_-+_ → ∞ | Extreme  ●●●●● | 0.0% |
|  |  |  |  |  |
| ***H1c:*** *‘not human-caused’* | | | | |
| Brazil | 7.3%  [6.1, 8.6] | BF_+-_ → ∞ | Extreme  ●●●●● | 0.0% |
| Canada | 4.8%  [3.7, 6.0] | BF_+-_ → ∞ | Extreme  ●●●●● | 0.0% |
| China | 7.1%  [6.0, 8.4] | BF_+-_ → ∞ | Extreme  ●●●●● | 0.0% |
| Germany | 7.4%  [6.0, 8.9] | BF_+-_ → ∞ | Extreme  ●●●●● | 0.0% |
| India | 2.9%  [1.5, 4.4] | BF_+-_ = 5,453.55 | Extreme  ●●●●● | 11.3% |
| Indonesia | -3.4%  [-4.7, -2.0] | BF_-+_ → ∞ | Extreme  ●●●●● | 6.9% |
| Italy | 6.5%  [5.3, 7.7] | BF_+-_ → ∞ | Extreme  ●●●●● | 0.0% |
| Japan | 4.9%  [3.7, 6.2] | BF_+-_ → ∞ | Extreme  ●●●●● | 0.0% |
| Mexico | 0.1%  [-1.2, 1.4] | BF_+-_ = 1.08 | Weak  ●○○○○ | 100.0% |
| Poland | 6.3%  [5.2, 7.6] | BF_+-_ → ∞ | Extreme  ●●●●● | 0.0% |
| Thailand | 6.0%  [4.7, 7.4] | BF_+-_ → ∞ | Extreme  ●●●●● | 0.0% |
|  |  |  |  |  |
| ***H1d:*** *‘not happening’* | | | | |
| Brazil | 8.0%  [6.6, 9.5] | BF_+-_ → ∞ | Extreme  ●●●●● | 0.0% |
| Canada | 9.4%  [8.1, 10.9] | BF_+-_ → ∞ | Extreme  ●●●●● | 0.0% |
| China | 6.2%  [5.1, 7.3] | BF_+-_ → ∞ | Extreme  ●●●●● | 0.0% |
| Germany | 9.2%  [8.0, 10.4] | BF_+-_ → ∞ | Extreme  ●●●●● | 0.0% |
| India | 7.7%  [6.2, 9.1] | BF_+-_ → ∞ | Extreme  ●●●●● | 0.0% |
| Indonesia | 9.4%  [8.1, 10.8] | BF_+-_ → ∞ | Extreme  ●●●●● | 0.0% |
| Italy | 8.5%  [7.3, 9.9] | BF_+-_ → ∞ | Extreme  ●●●●● | 0.0% |
| Japan | 6.0%  [4.8, 7.5] | BF_+-_ → ∞ | Extreme  ●●●●● | 0.0% |
| Mexico | 11.1%  [9.7, 12.5] | BF_+-_ → ∞ | Extreme  ●●●●● | 0.0% |
| Poland | 9.8%  [8.3, 11.2] | BF_+-_ → ∞ | Extreme  ●●●●● | 0.0% |
| Thailand | 6.8%  [5.6, 8.1] | BF_+-_ → ∞ | Extreme  ●●●●● | 0.0% |

| ***Combined:*** *‘mainly and partly human-caused’* | | | | |
| --- | --- | --- | --- | --- |
| Brazil | -20.8%  [-23.4, -18.2] | BF_-+_ → ∞ | Extreme  ●●●●● | 0.0% |
| Canada | -15.6%  [-18.0, -13.4] | BF_-+_ → ∞ | Extreme  ●●●●● | 0.0% |
| China | -12.6%  [-15.0, -10.2] | BF_-+_ → ∞ | Extreme  ●●●●● | 0.0% |
| Germany | -17.0%  [-19.4, -14.8] | BF_-+_ → ∞ | Extreme  ●●●●● | 0.0% |
| India | -12.0%  [-14.6, -9.5] | BF_-+_ → ∞ | Extreme  ●●●●● | 0.0% |
| Indonesia | -7.5%  [-10.1, -5.0] | BF_-+_ → ∞ | Extreme  ●●●●● | 0.0% |
| Italy | -14.7%  [-17.1, -12.3] | BF_-+_ → ∞ | Extreme  ●●●●● | 0.0% |
| Japan | -14.1%  [-16.6, -11.8] | BF_-+_ → ∞ | Extreme  ●●●●● | 0.0% |
| Mexico | -17.1%  [-19.6, -14.7] | BF_-+_ → ∞ | Extreme  ●●●●● | 0.0% |
| Poland | -19.7%  [-22.3, -17.3] | BF_-+_ → ∞ | Extreme  ●●●●● | 0.0% |
| Thailand | -16.4%  [-18.7, -14.2] | BF_-+_ → ∞ | Extreme  ●●●●● | 0.0% |

*Note.* The mean represents the posterior difference between the actual percentage of beliefs in the YouGov survey and the perceived percentages in this sample. Positive values indicate overestimation; negative values indicate underestimation. CrI = credible interval. BF_-+_ indicates one-sided testing of two competing hypotheses—that the actual percentage is underestimated (H_-_) *vs*. that the actual percentage is overestimated (H_+_). BF_-+_ = 10 would, therefore, mean that the data are ten times more likely under the H_-_ of underestimation than the H_+_ of overestimation, whereas BF_+-_ = 10 would indicate the opposite. The column ‘evidence’ categorizes the strength of evidence according to Jeffreys (1961) (Supplement E): Insufficient evidence: ○○○○○; weak evidence: ●○○○○; moderate evidence: ●●○○○; strong evidence: ●●●○○; very strong evidence: ●●●●○; extremely strong evidence: ●●●●●. Blue indicates evidence in favor of the tested hypothesis; red indicates evidence against it. The column ‘% inside region of practical equivalence (ROPE)’ indicates the proportion of the 95% equal-tailed credible interval of the posterior distribution that falls within the ROPE, defined as the percentage of each belief category from the YouGov survey ± the margins of error based on the 95% confidence interval to account for sampling uncertainty.

**Table F2**

*Summary of Pluralistic Ignorance Effects among Climate Change Believers across all Country*

| **Country** | **Mean pluralistic ignorance [90% CrI]** | **Bayes factor** | **Evidence in favor of / against Hypothesis** | **% inside region of practical equivalence**  **(ROPE)** |
| --- | --- | --- | --- | --- |
| ***Combined:*** *‘mainly and partly human-caused’ among believers* | | | | |
| Brazil | -14.5%  [-16.8, -12.2] | BF_-+_ → ∞ | Extreme  ●●●●● | 0.0% |
| Canada | -10.1%  [-12.0, -8.2] | BF_-+_ → ∞ | Extreme  ●●●●● | 0.0% |
| China | -9.2%  [-11.2, -7.1] | BF_-+_ → ∞ | Extreme  ●●●●● | 0.0% |
| Germany | -12.0%  [-13.9, -10.1] | BF_-+_ → ∞ | Extreme  ●●●●● | 0.0% |
| India | -4.9%  [-7.1, -2.7] | BF_-+_ = 5,999.00 | Extreme  ●●●●● | 1.2% |
| Indonesia | 2.0%  [-0.02, 4.1] | BF_+-_ = 15.16 | Strong  ●●●○○ | 72.3% |
| Italy | -9.8%  [-11.7, -7.8] | BF_-+_ → ∞ | Extreme  ●●●●● | 0.0% |
| Japan | -8.1%  [-10.1, -6.1] | BF_-+_ → ∞ | Extreme  ●●●●● | 0.0% |
| Mexico | -10.8%  [-13.0, -8.6] | BF_-+_ → ∞ | Extreme  ●●●●● | 0.0% |
| Poland | -13.4%  [-15.8, -11.2] | BF_-+_ → ∞ | Extreme  ●●●●● | 0.0% |
| Thailand | -10.0%  [-12.2, -7.9] | BF_-+_ → ∞ | Extreme  ●●●●● | 0.0% |

*Note.* The mean represents the posterior difference between the actual percentage of pro-climate beliefs in the YouGov survey and the perceived percentages of pro-climate beliefs among climate change believers in this sample. Positive values indicate overestimation; negative values indicate underestimation. CrI = credible interval. BF_-+_ indicates one-sided testing of two competing hypotheses—that the actual percentage is underestimated (H_-_) *vs*. that the actual percentage is overestimated (H_+_). BF_-+_ = 10 would, therefore, mean that the data are ten times more likely under the H_-_ of underestimation than the H_+_ of overestimation, whereas BF_+-_ = 10 would indicate the opposite. The column ‘evidence’ categorizes the strength of evidence according to Jeffreys (1961) (Supplement E): Insufficient evidence: ○○○○○; weak evidence: ●○○○○; moderate evidence: ●●○○○; strong evidence: ●●●○○; very strong evidence: ●●●●○; extremely strong evidence: ●●●●●. Blue indicates evidence in favor of the tested hypothesis; red indicates evidence against it. The column ‘% inside region of practical equivalence (ROPE)’ indicates the proportion of the 95% equal-tailed credible interval of the posterior distribution that falls within the ROPE, defined as the percentage of climate change believers from the YouGov survey ± the margins of error based on the 95% confidence interval to account for sampling uncertainty.

**Supplement G**

**Intervention Effects Across Countries**

**Figure G1**

**
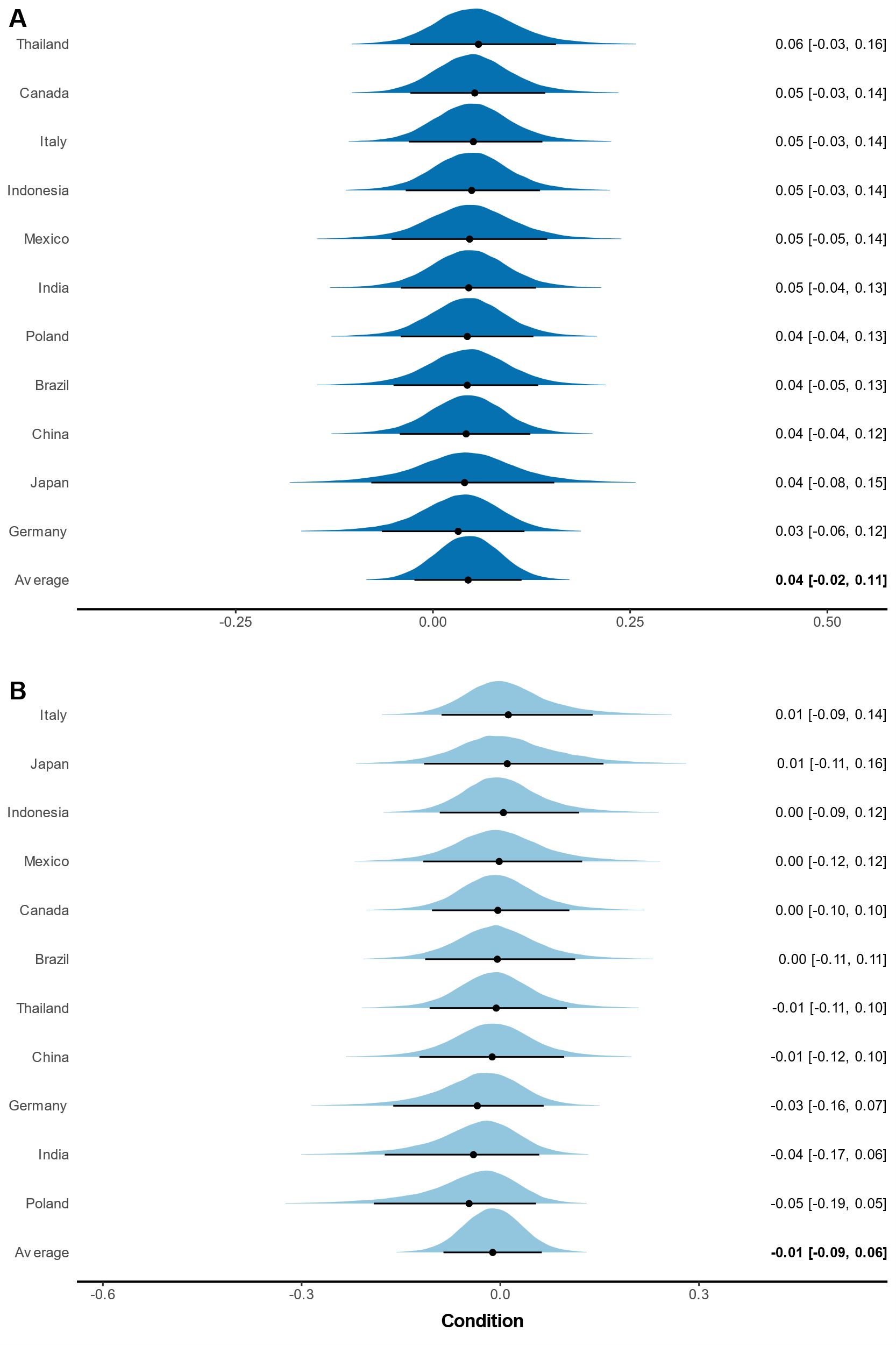
***Country-Level Intervention Effect on (A) Willingness to Express One’s Pro-Climate Opinion, (B) Willingness to Change One’s Lifestyle, (C) Prioritization of Government Action on Climate Change, and (D) Group Efficacy Beliefs*

**
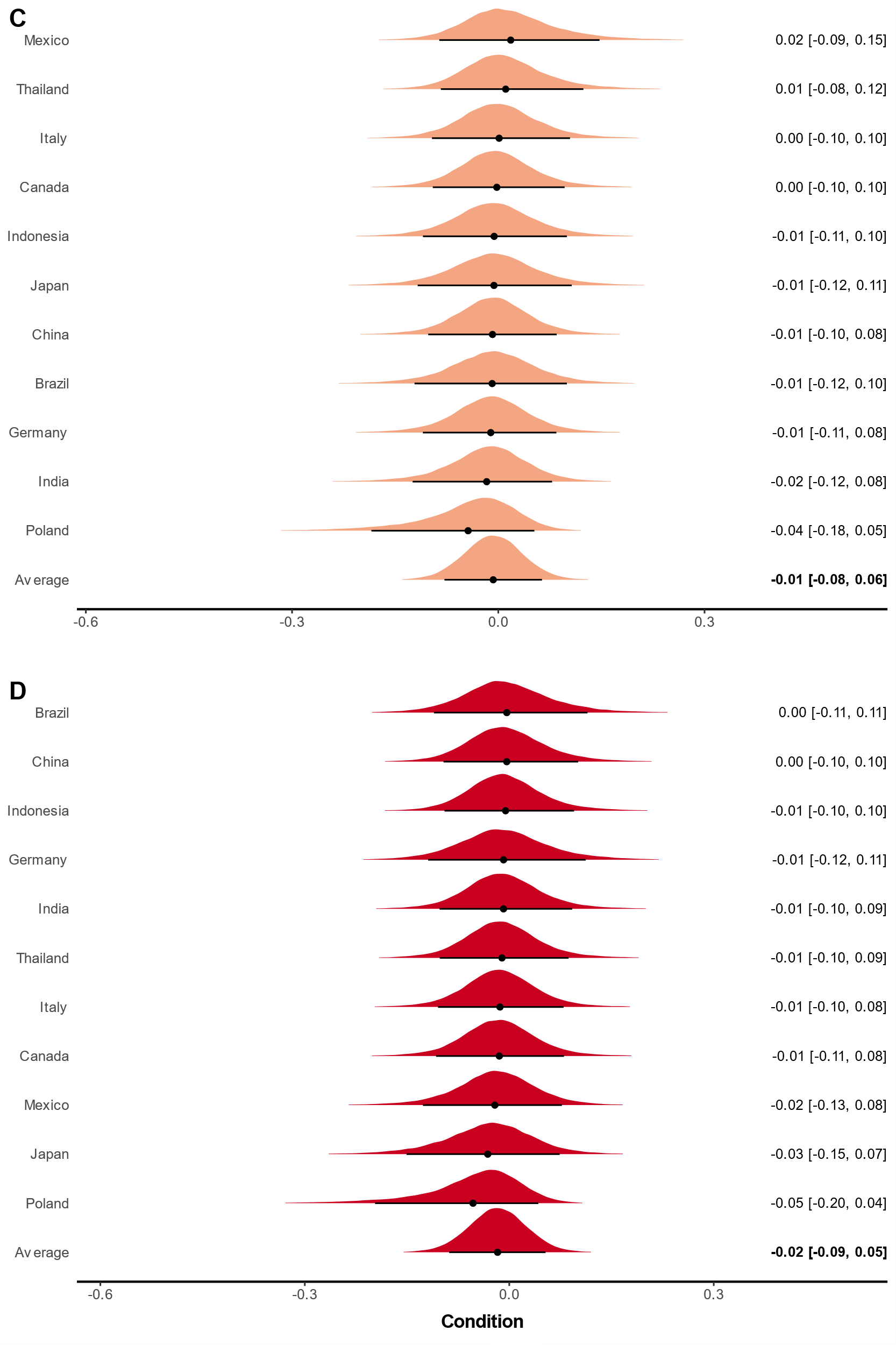
**

**Supplement H**

**Sensitivity and Exploratory Analyses**

As a robustness check, we re-ran all analyses without control variables (Table H1). These sensitivity analyses show similar results as the primary analyses, suggesting that the results are robust. We also conducted exploratory subgroup analyses to investigate the intervention effectiveness among the main target group, climate change believers who underestimated the public consensus in their country prior to being exposed to the intervention (*n* = 2,131-2,246). Consistent with the main findings, the intervention is largely ineffective even among this group (Table H2).

**Table H1**

*Results Without Control Variables*

| **Hypothesis /**  **Research question** | **Estimate  [90-95% CrI]** | **Bayes factor / p-value** | **Evidence in favor of / against Hypothesis** | **Comparison to main findings** |
| --- | --- | --- | --- | --- |
| **H3:** willingness to express one’s opinion on climate change | 0.04  [-0.03, 0.11] | 5.63 | Moderate ●●○○○ | Consistent in terms of direction and magnitude of the effect as well as the Bayes factor |
| **H4a:** personal willingness to change lifestyle | -0.02  [-0.10, 0.07] | 217.25 | Extremely strong ●●●●● | Consistent in terms of direction and magnitude of the effect as well as the Bayes factor |
| **H4b:** expectations about others’ willingness to change their lifestyle | 1.41% | .087 |  | Consistent in terms of direction and magnitude of the effect as well as *p*-value |
| **H5a:** personal support for government action | -0.01 [-0.10, 0.07] | 235.76 | Extremely strong ●●●●● | Consistent in terms of direction and magnitude of the effect as well as the Bayes factor |
| **H5b:** expectations about others’ government support | 1.35% | .094 |  | Consistent in terms of direction and magnitude of the effect as well as *p*-value |
| **RQ2:** group efficacy beliefs | -0.02  [-0.11, 0.07] | 211.74 | Extremely strong ●●●●● | Consistent in terms of direction and magnitude of the effect as well as the Bayes factor |

*Note.* CrI = credible interval. Insufficient evidence: ○○○○○; weak evidence: ●○○○○; moderate evidence: ●●○○○; strong evidence: ●●●○○; very strong evidence: ●●●●○; extremely strong evidence: ●●●●●. Blue indicates evidence in favor of the tested hypothesis or research question; red indicates evidence against it.

**Table H2**

*Effectiveness of the Intervention Among Climate Change Believers Who Underestimated the Public Consensus Prior to the Intervention*

| **Hypothesis /**  **Research question** | **Estimate  [90-95% CrI]** | **Bayes factor / p-value** | **Evidence in favor of / against Hypothesis** | **Comparison to main findings** |
| --- | --- | --- | --- | --- |
| **H3:** willingness to express one’s opinion on climate change | 0.03  [-0.06, 0.11] | 2.45 | Weak ●○○○○ | Consistent in terms of direction and magnitude of the effect but slightly lower Bayes factor |
| **H4a:** personal willingness to change lifestyle | -0.02  [-0.14, 0.11] | 158.48 | Extremely strong ●●●●● | Consistent in terms of direction and magnitude of the effect as well as the Bayes factor |
| **H4b:** expectations about others’ willingness to change their lifestyle | 1.83% | .049 |  | Slightly—although not meaningfully—larger and now significant effect |
| **H5a:** personal support for government action | -0.01 [-0.12, 0.10] | 186.19 | Extremely strong ●●●●● | Consistent in terms of direction and magnitude of the effect as well as the Bayes factor |
| **H5b:** expectations about others’ government support | 2.36% | .009 |  | Slightly—although not meaningfully—larger and now significant effect |
| **RQ2:** group efficacy beliefs | -0.05  [-0.16, 0.06] | 134.44 | Extremely strong ●●●●● | Consistent in terms of direction and magnitude of the effect as well as the Bayes factor |

*Note.* CrI = credible interval. Insufficient evidence: ○○○○○; weak evidence: ●○○○○; moderate evidence: ●●○○○; strong evidence: ●●●○○; very strong evidence: ●●●●○; extremely strong evidence: ●●●●●. Blue indicates evidence in favor of the tested hypothesis or research question; red indicates evidence against it.

**Supplement I**

**Details on the Follow-up Study in Brazil**

**Participants**

The cross-quota samples based on age and sex were collected through *besample* (https://besample.app/) from January 30 to February 29, 2024. Participants were paid for a 10-minute survey according to the minimum wage in Brazil. Inclusion and exclusion criteria were identical to the main study.

A total of 371 participants started the online survey experiment. Of these, 128 (34.5%) were screened out because they did not complete the survey experiment (*n* =16, 4.3%), they were not eligible for this study (*n* = 24, 6.5%), failed the attention check (*n* = 60, 16.2%), completed the survey experiment in under 3 minutes (*n* = 26, 7.0%), or were potential bots (*n* = 2, 0.5%). Moreover, as in the main experimental study, we excluded those who did not believe in partly or mainly human-caused climate change (*n* = 24, 6.5%). These exclusions resulted in an analytic sample of 229 climate change believers across 11 countries. Participants were, on average, 31.5 years old (*SD* = 9.5; range: 18-63 years), 139 (63.5%) were female, and 142 (64.8%) held a university degree. Most participants (*n* = 196; 89.5%) lived in urban areas.

**Materials**

The materials and procedure were identical to those in the main study, with two exceptions. First, we measured updated beliefs directly after message exposure: “What percentage of Brazilians, do you believe, would think the following ways about climate change? Please indicate a number from 0% (no one) to 100% (everyone).” Participants indicated their answer on a slider from 0% to 100%. Second, we added a memory check at the end of the survey before the demographics block in the intervention condition: “Earlier in this study, you saw a message about a recent survey with information about the percentage (%) of Brazilians who believe that the climate is changing and human activity is partly or mainly responsible. To the best of your recollection, what was the exact number that was presented to you? Note: Please enter only the number, without the % sign.”

**Data Analysis**

We used frequentist fractional logistic regression models, with post-intervention beliefs about others’ pro-climate change beliefs as a bounded outcome. We used condition as a binary predictor and pre-intervention beliefs about others’ pro-climate change beliefs as a continuous predictor.
